# Genome-Wide Identification, Evolution, and Expression Analysis of the ATP-Binding Cassette Transporter Gene Family in *Brassica rapa*

**DOI:** 10.3389/fpls.2017.00349

**Published:** 2017-03-17

**Authors:** Chao Yan, Weike Duan, Shanwu Lyu, Ying Li, Xilin Hou

**Affiliations:** ^1^State Key Laboratory of Crop Genetics and Germplasm Enhancement, Key Laboratory of Biology and Germplasm Enhancement of Horticultural Crops in East China, Ministry of Agriculture, Nanjing Agricultural UniversityNanjing, China; ^2^School of Life Science and Food Engineering, Huaiyin Institute of TechnologyHuaian, China

**Keywords:** *Brassica rapa*, ATP-binding cassette transporter, phylogenetic analysis, whole genome triplication, gene expression, evolutionary fate

## Abstract

ATP-binding cassette (ABC) proteins can act as transporters of different substrates across biological membranes by hydrolyzing ATP. However, little information is available about ABC transporters in *Brassica rapa*, an important leafy vegetable. In the present study, we carried out genome-wide identification, characterization and molecular evolution analyses of ABC gene family in *B. rapa* and 9 other plant species. A total of 179 *B. rapa* ABC genes (BraABCs) were identified. Among them, 173 BraABCs were identified on 10 chromosomes. Based on phylogenetic analysis and domain organization, the BraABC family could be grouped into eight subfamilies. BraABCs in the same subfamily showed similar motif composition and exon-intron organization. Common and unique *cis*-elements involved in the transcriptional regulation were also identified in the promoter regions of BraABCs. Tissue-expression analysis of BraABCs demonstrated their diverse spatiotemporal expression profiles. Influences of the whole genome triplication (WGT) on the evolution of BraABCs were studied in detail. BraABCs were preferentially retained compared with their neighboring genes during diploidization after WGT. Synteny analysis identified 76 pairs of syntenic BraABC paralogs among the three subgenomes of *B. rapa*, and 10 paralog pairs underwent positive selection with ω (= *Ka*/*Ks*) ratios greater than 1. Analyses of the expression patterns of syntenic BraABC paralogs pairs across five tissues and under stress treatments revealed their functional conservation, sub-functionalization, neo-functionalization and pseudogenization during evolution. Our study presents a comprehensive overview of the ABC gene family in *B. rapa* and will be helpful for the further functional study of BraABCs in plant growth, development, and stress responses.

## Introduction

ATP-binding cassette (ABC) transporters constitute one of the largest gene families that are ubiquitously present in all living organisms, from prokaryotes to humans (Dassa and Bouige, 2001). The majority of ABC proteins that have been characterized are ATP-dependent, and membrane-bound transporters are able to translocate a wide range of molecules through intra- and extracellular membranes (Higgins, 1992). Plant genomes are characterized by a large number of ABC genes (ABCs) encoding for more than 100 ABC transporters, which is more than most other organisms, that are involved in a broad range of biological functions (Kang et al., 2011).

The possession of a nucleotide-binding domain (NBD) is usually used to define ABC proteins (Verrier et al., 2008). There are several highly conserved motifs within the NBD domain, including the Walker A and Walker B sequences, the ABC signature motif, the H loop and the Q loop (Higgins and Linton, 2004). Apart from the NBD domain, ABC proteins also contain hydrophobic transmembrane domains (TMDs). Several transmembrane α-helices are generally present in TMDs. NBDs usually act as energy providers for substrate translocation or non-transport processes by ATP-binding and ATP- hydrolyzing. TMDs function as recognizers and channels for substrates to translocate the lipid bilayer (Sánchez-Fernández et al., 2001). In eukaryotes, there are two common arrangements for ABC transporters: full-sized transporters and half-sized transporters. The structure of canonical ABC transporters (full-sized ABCs) contains four domains: two NBDs and two TMDs in a single polypeptide. Half-sized transporters consist of only two domains (1 TMD and 1 NBD). Half-sized ABCs have to form homo- or heterodimers to conduct the function of the substrate pump. ABC proteins lacking a TMD are usually not involved in transmembrane transport.

Several categorization methods have been proposed to classify ABC proteins, of which the Human Genome Organization (HUGO) scheme has currently been widely adopted to classify human and plant ABC proteins (Dean et al., 2001; Verrier et al., 2008). According to the HUGO scheme, eukaryotic ABC proteins are divided into eight subfamilies based on the NBD phylogenetic relationship, homologous relationship and domains organization: from ABC subfamily A (ABCA) to ABC subfamily H (ABCH). ABCH was identified in arthropod genomes, but is absent in fungi, mammals and plants (Annilo et al., 2006; Dermauw and Van Leeuwen, 2014). Recently, ABC subfamily I (ABCI) containing the “prokaryotic”-type ABCs was found in plants but is absent in most animal genomes (Verrier et al., 2008). In total, nine subfamilies (ABCA-ABCI) have been identified, and eight of them (ABCA-ABCG and ABCI) are present in plant genomes (Kang et al., 2011). Proteins of the ABCA-ABCD subfamilies have a forward direction for domains organization (TMD-NBD). Relatively, the proteins of the ABCG and ABCH subfamilies contain the reverse domain organization (NBD-TMD). While ABCE and ABCF proteins consist of only two NBDs, these proteins are characterized as soluble proteins. ABCI proteins usually possess only one single domain, mainly NBD or TMD. The genetic evidence indicates that some of these single domains encoded by ABCI proteins can assemble into functional multi-subunit ABC transporters (Verrier et al., 2008).

The functions of ABC proteins in humans have been extensively studied. In addition to acting as active transporters, some ABCs function as ion channels, receptors or are even involved in mRNA translation and ribosome biogenesis. In humans, ABCs are also identified as having multidrug resistances in cancer cells; for example, proteins within the ABCB and ABCG subfamilies can protect cells from hydrophobic xenobiotics (Sarkadi et al., 2006). In plants, ABCs were initially identified as transporters participating in detoxification processes (Martinoia et al., 1993). Subsequently, an increasing number of plant ABCs have been functionally characterized as far away from detoxification functions. Plant ABCs participate in many important physiological and developmental processes; furthermore, the transport substrates of plant ABCs are divergent and include conjugated compounds, phytohormones, primary products, lipids and lipophilic compounds (Kang et al., 2011).

The evolution of the angiosperm genome is characterized by polyploidization through whole-genome duplication (WGD), followed by diploidization, which is typically accompanied by considerable homoeologous gene loss (Stebbins, 1950). A number of polyploidy events have been identified along different lineages of flowering plants. After polyploidy, duplicated genes experience different fates over evolutionary time. Most likely, one copy of paralogous genes loses or becomes pseudogenes (Lynch and Conery, 2000, 2003). However, numerous functional importance duplicated gene pairs can survive by undergoing tens of millions of years of natural selection (Schnable et al., 2009; Schmutz et al., 2010). Gene categories whose products include transcription factors, ribosomal proteins or protein kinases are preferentially retained (Blanc and Wolfe, 2004; Maere et al., 2005). This can be explained by the gene dosage hypothesis, positing that genes whose products interact with one another or in networks tend to be dosage sensitive and would be over-retained (Thomas et al., 2006; Birchler and Veitia, 2007). In sum, duplicated genes undergo one of the following evolutionary fates: functional conservation (in which both copies maintain the same function), sub-functionalization (the ancestral function is subdivided between copies), neo-functionalization (one copy acquires a new function) or pseudogenization (one copy becomes unexpressed or functionless) (Lynch and Conery, 2000).

Brassicaceae is a large plant family containing ~338 genera and 3,700 species (Bailey et al., 2006). Owing to its remarkable species, genetic and physiological diversity, as well as significant economic potential, Brassicaceae has become a model for polyploidy and evolutionary studies. In Brassicaceae, *Arabidopsis* underwent three WGD events after its lineage diverged from the monocot lineage: the paleo-hexaploidy (γ) duplication shared with most dicots and two subsequent genome duplications (α and β) since its divergence from *Carica papaya* (Bowers et al., 2003). *B. rapa*, a diploid *Brassica* species, not only shared the three paleo-polyploidy events with *Arabidopsis* but also underwent a further whole genome triplication (WGT) since its divergence from *Arabidopsis* 13 to 17 million years ago (Town et al., 2006). After the WGT, *B. rapa* has undergone considerable fractionation: the 41,174 genes in the *B. rapa* genome are considerably fewer than would be expected from a simple WGT of the ~27,000 genes in the *Arabidopsis* genome (Wang et al., 2011). The extent of gene loss varies among the three subgenomes. The LF (least fractionated) subgenome retains ~70% of the genes found in *Arabidopsis*, whereas the MF1 (medium fractionated) and MF2 (most fractionated) subgenomes retain ~46 and 36%, respectively (Wang et al., 2011). *B. rapa*, because of its close relationship with *Arabidopsis* and representative WGT event, has thus become an excellent model for studying the evolution of genome structure and gene fate after WGD.

As a superfamily, ~22 out of 130 *Arabidopsis* ABCs (AtABCs) have been functionally characterized in the model plant. *B. rapa* is an important leafy vegetable in the Brassicaceae family; however, little information about the ABC gene family is available in *B. rapa*. The objective of the present study was to conduct a genome-wide identification and characterization of the ABC gene family in *B. rapa*. We carried out comprehensive studies of the ABC gene family in *B. rapa* for the first time, including the phylogenetic relationships, chromosomal locations, gene structures, *cis*-elements, and expression profiles of BraABCs in different tissues. Influences of WGT on the evolution of BraABCs were also analyzed, including the retention and conservation of BraABCs after WGT and syntenic BraABC paralog pairs among three subgenomes of *B. rapa*. Expression patterns of syntenic paralog BraABCs in different tissues and under various abiotic stresses were also determined to investigate their molecular evolutionary fates. Our findings provide a basis for better understanding of the function and evolutionary history of BraABCs and will help to further investigate the detailed molecular and biological functions of BraABCs.

## Materials and methods

### Data sources and ABC sequences retrieval

The *B. rapa* annotated genome sequences were downloaded from the *Brassica* database (BRAD; http://brassicadb.org/brad/; Cheng et al., 2011). Several datasets and multiple steps were used to conduct a whole-genome search. Hmmsearch from the HMMER program (3.0) (Eddy, 1998) was used to search the *Brassica rapa* genome to identify putative ABCs containing the ABC-transporter domain (PF00005), the ABC-2 transporter domain (PF01061), the ABC transporter transmembrane region domain (PF00664), the CYT domain (PF01458) or the mce related protein domain (PF02470) at a threshold of *E* < 1E^−4^. The Hidden Markov Model (HMM) profiles of these domains were downloaded from the Pfam 27.0 database (http://Pfam.sanger.ac.uk/; Finn et al., 2013). Then, we manually verified the candidate ABCs using the SMART database (http://smart.embl-heidelberg.de/; Letunic et al., 2012), and sequences with obvious errors and/or lengths of less than 100 amino acids were manually removed.

### Multiple alignment and phylogenetic analysis of the ABC gene family

Amino acid sequences of the ABC-transporter domain (PF00005) were extracted according to their locations in ABC proteins. Multiple alignments of ATPase domain sequences were conducted using ClustalW (Thompson et al., 1997) of the MEGA6 (Tamura et al., 2013) software with the default options. The phylogenetic trees were constructed using the maximum likelihood (ML) method in MEGA6 with the Jones, Taylor, and Thornton (JTT) amino acid substitution model. One thousand bootstrap replications were performed in each analysis. The remaining parameters were set to the defaults.

### Conserved motifs identification and exon-intron structure analysis

Conserved motifs of ABC proteins were identified using the local MEME software version 4.9.0 (Bailey et al., 2009), with the following parameter settings: maximum number of motifs = 15, and optimum motif width = 10–200 residues; the other parameters were the defaults. The coding sequences (CDSs) and the DNA sequences were used to predict the exon-intron structure of ABCs using the Gene Structure Display Server 2.0 (GSDS 2.0) (http://gsds.cbi.pku.edu.cn; Hu et al., 2015).

### *Cis*-elements analysis and proteins interaction network prediction

All of the promoter sequences (2,000 bp upstream of initiation codon “ATG”) of BraABCs were extracted from the *B. rapa* genome according to the Generic File Format (GFF) file. Then, the *cis*-regulatory elements of promoters for each gene were identified by PLACE Web Signal Scan-PLACE (https://sogo.dna.affrc.go.jp/cgi-bin/sogo.cgi?lang=en&pj=640&action=page&page=newplace). The interaction network of BraABC proteins was constructed by applying STRING software (Search Tool for the Retrieval of Interacting Genes/Proteins, http://string-db.org/).

### Gene chromosomal location, gene synteny analysis, and syntenic BraABCs paralogs pair identification

Chromosomal locations of BraABCs were drawn from top to bottom on *B. rapa* chromosomes according to their positions in genome annotation. The Perl in-house program was used to draw the location images of BraABCs on chromosomes. Syntenic relationships of ABCs between *Arabidopsis* and *B. rapa* were identified by searching “syntenic gene” in the *Brassica* database (BRAD) (Cheng et al., 2011). Then, the Circos program (Krzywinski et al., 2009) was applied to present the syntenic relationships on their chromosomes.

After the identification of the BraABCs, syntenic BraABCs paralogs pairs among the three *B. rapa* subgenomes were identified by searching “syntenic gene” in BRAD (Cheng et al., 2011). The non-synonymous substitution rate (*Ka*), the synonymous substitution rate (*Ks*) and the ω (= *Ka*/*Ks*) of paralog pairs were estimated by KaKs_Calculator 2.0 (Wang et al., 2010). The duplication date of paralog pairs was estimated according to formula T = *Ks*/2λ, assuming a clock-like rate (λ) of 1.5 synonymous substitutions per 10^8^ years for *B. rapa* (Koch et al., 2000).

### Expression pattern analysis

To analyze the expression patterns of BraABCs in different tissues, we used the reported Illumina RNA-seq data by Tong et al. (2013). Of the dataset, five tissues, including the roots, stems, leaves, flowers and siliques of *B. rapa* accession *Chiifu-401-42* were analyzed. Gene expression levels were quantified by FPKM (Fragments Per Kilobase of transcript per Million fragments mapped) values. An expression cluster of BraABCs in the five tissues was generated by Cluster 3.0 (http://bonsai.hgc.jp/~mdehoon/software/cluster/software.htm), and the cluster results were shown using Tree View software (http://jtreeview.sourceforge.net/). The RNA-seq dataset was also used to analyze the expression profiles of syntenic paralog pairs in different tissues.

To investigate the expression patterns of syntenic BraABC paralog pairs under normal and abiotic stress conditions, the cultivar *Chiifu-401-42* was cultivated in potting soil at 24/16°C, with a photoperiod of 14/10 h for day/night in a growth chamber. Five-leaf-stage plants were used to conduct different abiotic stress treatments under continuous time (6, 12, and 24 h). Hormone treatment was performed with 100 μM ABA; for drought stress treatment, the pots were irrigated with 15% (w/v) polyethylene glycol PEG and kept standing in the irrigation solution for 30 min under normal growth conditions; salt treatment was performed with 250 mM NaCl. After the treatments, the total RNAs were isolated from young leaves using an RNA kit (TaKaRa, Dalian, China). The RNAs were reverse transcribed into cDNA with a PrimeScript RT reagent Kit (TaKaRa, Dalian, China). Six pairs of syntenic paralogs were randomly selected to conduct qRT-PCR. The *B. rapa* actin gene Bra028615 was used as an internal control. Primers for qRT-PCR were designed using Beacon Designer 7. The qRT-PCR experiments were performed with three biological and technical replicates. The SYBR Premix Ex Taq kit® (TaKaRa, Dalian, China) was used to detect gene expression according to the manufacturer's recommendations on the One-step Real-Time PCR System Time PCR Detection System (Applied Biosystems, Foster City, CA, USA). The cycling profile was as follows: 94°C for 30 s, 94°C for 10 s for 40 cycles, and 58°C for 30 s, followed by a melting curve analysis at 65°C for 10 s for 61 cycles. The relative gene expression levels were analyzed using the comparative Ct value method (Heid et al., 1996).

## Results

### Genome-wide identification, phylogenetic analysis, and classification of ABC genes in *B. rapa*

To explore all of the putative ABCs in the *B. rapa* genome, the HMM profiles of the ABC-transporter domain, the ABC-2 transporters domain, the ABC transporter transmembrane region domain, the CYT domain and the mce related protein domain were employed as queries to search against the protein database of *B. rapa* using the Hmmsearch program. A total of 179 BraABCs, which contained at least one of the above domains, were identified (Table 1). Among the 179 BraABC proteins, 162 proteins contained one or more NBD domains, and an additional 17 proteins without NBD domains were assigned to ABCI, based on the domain composition of the ABC proteins (Verrier et al., 2008).

**Table 1:** **Basic information about the ABC transporter gene family in ***B. rapa*****.

**Gene**	**Locus name**	**Subfamily**	**Chromosome**	**Position**	**Length (aa)**	**Exon number**	**MW (kDa)**	**PI**	**TMs**
*BraABCA1*	Bra033785	ABCA	A01	14252485–14257069	942	16	105.64	8.62	7
*BraABCA2*	Bra000236	ABCA	A03	10073306–10085122	1842	40	204.53	6.66	13
*BraABCA3*	Bra012931	ABCA	A03	21458462–21462337	529	10	65.54	7.98	2
*BraABCA4*	Bra012928	ABCA	A03	21470698–21474908	927	15	102.56	8.37	6
*BraABCA5*	Bra012927	ABCA	A03	21475917–21479405	615	10	67.56	8.99	6
*BraABCA6*	Bra018113	ABCA	A06	10311197–10315562	942	16	105.32	8.2	7
*BraABCA7*	Bra018114	ABCA	A06	10319900–10323172	629	10	71.59	8.78	2
*BraABCA8*	Bra018115	ABCA	A06	10325041–10329173	926	15	104.29	9.14	7
*BraABCA9*	Bra018116	ABCA	A06	10341780–10346225	986	15	108.65	7.07	6
*BraABCA10*	Bra019513	ABCA	A06	12666176–12670670	893	18	100.88	9.16	5
*BraABCA11*	Bra015049	ABCA	A07	3961325–3965509	893	15	100.58	8.97	5
*BraABCB1*	Bra011043	ABCB	A01	4272644–4275279	687	3	76.09	9.63	5
*BraABCB2*	Bra013936	ABCB	A01	8540846–8546690	1234	12	135.54	9.34	12
*BraABCB3*	Bra033043	ABCB	A02	21713331–21718585	1244	7	135.72	8.06	11
*BraABCB4*	Bra006776	ABCB	A03	5011490–5015371	724	16	80.12	9.78	5
*BraABCB5*	Bra023087	ABCB	A03	8624046–8629351	1339	10	146.73	7.63	12
*BraABCB6*	Bra000136	ABCB	A03	9481070–9486731	1403	11	154.91	6.33	12
*BraABCB7*	Bra012621	ABCB	A03	23170309–23175454	1247	12	134.94	7.06	11
*BraABCB8*	Bra019135	ABCB	A03	26140733–26146345	1241	13	136.31	7.93	10
*BraABCB9*	Bra014756	ABCB	A04	2832718–2839592	1400	11	154.27	5.96	11
*BraABCB10*	Bra028221	ABCB	A04	6791848–6795123	641	16	69	8.73	5
*BraABCB11*	Bra017216	ABCB	A04	15986960–15992620	1338	10	146.67	7.49	10
*BraABCB12*	Bra040475	ABCB	A04	18946035–18950787	1287	10	138.86	6.96	9
*BraABCB13*	Bra004484	ABCB	A05	370135–375843	1284	10	138.67	6.69	9
*BraABCB14*	Bra005036	ABCB	A05	3097621–3103960	1408	11	155.55	6.08	11
*BraABCB15*	Bra019907	ABCB	A06	3774311–3781400	1225	13	134.18	7.17	10
*BraABCB16*	Bra025425	ABCB	A06	21585065–21593092	1241	9	136.65	8.6	9
*BraABCB17*	Bra025359	ABCB	A06	22003874–22010062	1252	9	136.75	8.16	10
*BraABCB18*	Bra025331	ABCB	A06	22120036–22124400	1139	5	124.69	9.1	8
*BraABCB19*	Bra025328	ABCB	A06	22135599–22140199	1069	4	117.26	9.15	6
*BraABCB20*	Bra025326	ABCB	A06	22155393–22160709	1244	7	135.98	8.31	11
*BraABCB21*	Bra003445	ABCB	A07	13221037–13227556	1254	11	136.3	7.11	11
*BraABCB22*	Bra003950	ABCB	A07	15930698–15934778	699	17	77.98	9.21	4
*BraABCB23*	Bra003490	ABCB	A07	16736177–16741372	1292	10	139.66	6.55	11
*BraABCB24*	Bra010464	ABCB	A08	14658571–14663250	683	15	74.27	9.58	5
*BraABCB25*	Bra030503	ABCB	A08	21539124–21543766	1276	10	137.17	7.76	9
*BraABCB26*	Bra039056	ABCB	A09	1349788–1355315	690	4	75.74	9.81	5
*BraABCB27*	Bra039055	ABCB	A09	1364841–1379565	1415	13	155.12	7.2	8
*BraABCB28*	Bra039042	ABCB	A09	1451543–1458438	1252	9	136.71	8.59	10
*BraABCB29*	Bra032864	ABCB	A09	12328042–12339538	1228	9	134.18	9.61	11
*BraABCB30*	Bra032856	ABCB	A09	12379011–12383707	1239	9	134.36	9.35	12
*BraABCB31*	Bra032855	ABCB	A09	12384484–12384840	118	1	12.87	10.28	0
*BraABCB32*	Bra027534	ABCB	A09	13505446–13513218	1224	7	133.68	8.47	9
*BraABCB33*	Bra017539	ABCB	A09	16196016–16200573	1031	11	112.89	8.99	11
*BraABCB34*	Bra017540	ABCB	A09	16208131–16208877	216	2	23.56	6.03	0
*BraABCB35*	Bra033331	ABCB	A10	4205096–4210047	1266	10	136.17	7.82	9
*BraABCB36*	Bra002664	ABCB	A10	8338410–8342848	722	17	79.74	9.83	5
*BraABCB37*	Bra002094	ABCB	A10	11533544–11539264	1231	11	133.9	7.68	10
*BraABCB38*	Bra009500	ABCB	A10	16942154–16944882	633	9	68.89	10.07	2
*BraABCC1*	Bra023893	ABCC	A01	20873809–20879204	1447	11	160.61	7.36	14
*BraABCC2*	Bra001488	ABCC	A03	16641078–16647142	1493	8	165.31	6.38	14
*BraABCC3*	Bra001490	ABCC	A03	16652692–16657965	1479	8	163.98	6.37	14
*BraABCC4*	Bra012617	ABCC	A03	23184921–23191874	1430	34	159.84	7.71	14
*BraABCC5*	Bra005402	ABCC	A05	5277561–5285323	1626	25	182.29	6.16	14
*BraABCC6*	Bra032248	ABCC	A05	12374900–12379965	1441	10	161.25	7.82	16
*BraABCC7*	Bra031267	ABCC	A05	16535804–16541428	1464	12	161.77	7.24	14
*BraABCC8*	Bra034706	ABCC	A05	21060822–21066705	1473	9	165.09	6.73	14
*BraABCC9*	Bra018722	ABCC	A06	2328689–2334077	1518	8	169.98	7.92	17
*BraABCC10*	Bra014879	ABCC	A07	5640904–5648508	1512	26	169.85	8.9	10
*BraABCC11*	Bra003402	ABCC	A07	13033974–13039675	1494	10	167.28	7.89	15
*BraABCC12*	Bra010773	ABCC	A08	16342602–16351477	1590	26	178.4	6.7	14
*BraABCC13*	Bra036682	ABCC	A09	5678744–5684163	1439	10	159.56	6.84	12
*BraABCC14*	Bra032386	ABCC	A09	21844669–21853187	1444	22	161.84	6.81	12
*BraABCC15*	Bra032385	ABCC	A09	21859808–21868932	1623	27	182.28	6.53	14
*BraABCC16*	Bra007434	ABCC	A09	29185025–29191292	1451	11	162.27	7.94	10
*BraABCC17*	Bra007695	ABCC	A09	30432248–30437756	1537	9	171.27	8.41	15
*BraABCC18*	Bra015291	ABCC	A10	2670172–2675455	1508	9	167.65	7	12
*BraABCC19*	Bra039348	ABCC	Scaffold000164	42040–47798	1463	8	163.82	6.42	12
*BraABCC20*	Bra039368	ABCC	Scaffold000164	156063–161062	1477	8	164.53	6.65	16
*BraABCC21*	Bra039369	ABCC	Scaffold000164	161873–166792	1437	8	161.31	7.23	12
*BraABCD1*	Bra033548	ABCD	A01	11206302–11209035	693	8	78.75	7.8	4
*BraABCD2*	Bra010652	ABCD	A08	15787520–15795700	1433	27	160.49	8.97	2
*BraABCE1*	Bra013385	ABCE	A01	5318724–5321407	581	11	65.6	8.32	0
*BraABCE2*	Bra021494	ABCE	A01	24963925–24966374	603	9	68.21	8.15	0
*BraABCE3*	Bra001515	ABCE	A03	16793599–16796264	603	9	67.88	8.33	0
*BraABCE4*	Bra012553	ABCE	A03	23587642–23590415	605	11	68.45	7.58	0
*BraABCE5*	Bra017243	ABCE	A04	15778643–15785578	757	15	84.08	6.4	0
*BraABCE6*	Bra020966	ABCE	A08	10894669–10897411	605	11	68.39	7.76	0
*BraABCE7*	Bra032841	ABCE	A09	12469694–12475157	456	7	51.42	8.52	0
*BraABCF1*	Bra023575	ABCF	A02	4001828–4003954	596	4	66.88	6.27	0
*BraABCF2*	Bra031898	ABCF	A02	27222260–27225249	687	5	77.16	6.66	0
*BraABCF3*	Bra006648	ABCF	A03	4464031–4466477	605	4	68.25	6.52	0
*BraABCF4*	Bra014809	ABCF	A04	3297356–3299134	592	1	65.93	5	0
*BraABCF5*	Bra027728	ABCF	A09	6603238–6607304	762	16	84.5	6.14	0
*BraABCF6*	Bra007079	ABCF	A09	27184498–27186654	718	1	80.11	6.04	0
*BraABCF7*	Bra009072	ABCF	A10	15137399–15140335	674	8	76.11	7.5	0
*BraABCG1*	Bra013916	ABCG	A01	8428094–8429815	573	1	63.89	9.47	6
*BraABCG2*	Bra026352	ABCG	A01	9839191–9841673	638	4	70.99	8.66	6
*BraABCG3*	Bra021173	ABCG	A01	23581848–23590158	1413	19	160.02	8.04	13
*BraABCG4*	Bra028729	ABCG	A02	2341235–2346157	752	10	82.82	9.45	6
*BraABCG5*	Bra022614	ABCG	A02	8526894–8528663	589	1	65.72	9.56	7
*BraABCG6*	Bra007999	ABCG	A02	12431267–12436434	659	4	72.91	9.29	7
*BraABCG7*	Bra026514	ABCG	A02	19834330–19837326	668	5	74.66	9.36	6
*BraABCG8*	Bra028945	ABCG	A03	5542028–5547011	1147	14	127.51	8.77	4
*BraABCG9*	Bra023065	ABCG	A03	8472384–8479166	1429	19	161.73	7.51	12
*BraABCG10*	Bra000469	ABCG	A03	11249683–11255770	1404	20	157.39	9.24	11
*BraABCG11*	Bra013141	ABCG	A03	20225924–20227822	632	1	70.96	9.59	5
*BraABCG12*	Bra012797	ABCG	A03	22187168–22194238	1371	21	155.08	7.85	13
*BraABCG13*	Bra019051	ABCG	A03	26553817–26556403	637	4	70.86	9.64	6
*BraABCG14*	Bra014773	ABCG	A04	2952295–2954552	710	2	79.25	7.02	7
*BraABCG15*	Bra014774	ABCG	A04	2977504–2983724	633	4	70.12	8.53	3
*BraABCG16*	Bra014775	ABCG	A04	3002630–3004729	699	1	77.75	8.29	6
*BraABCG17*	Bra014776	ABCG	A04	3021734–3028035	1284	2	143.14	9.37	11
*BraABCG18*	Bra033441	ABCG	A04	4220746–4230833	1132	17	127.34	6.65	5
*BraABCG19*	Bra034314	ABCG	A04	11848732–11855032	1420	23	161.65	8.34	13
*BraABCG20*	Bra034385	ABCG	A04	12263721–12272343	689	10	76.15	8.72	6
*BraABCG21*	Bra021598	ABCG	A04	13305142–13311183	1429	20	160.31	8.51	11
*BraABCG22*	Bra017241	ABCG	A04	15804449–15818281	2270	36	257.03	6.09	13
*BraABCG23*	Bra017198	ABCG	A04	16083910–16091064	1472	22	167.13	8.24	13
*BraABCG24*	Bra017193	ABCG	A04	16099619–16101847	742	1	82.05	9.41	6
*BraABCG25*	Bra005048	ABCG	A05	3166613–3168847	744	1	82.44	9.36	7
*BraABCG26*	Bra005051	ABCG	A05	3177024–3178868	614	1	67.92	10.13	7
*BraABCG27*	Bra005207	ABCG	A05	4010569–4016591	1423	22	161.28	6.92	13
*BraABCG28*	Bra005208	ABCG	A05	4017374–4024058	1415	22	160.09	8.32	13
*BraABCG29*	Bra005224	ABCG	A05	4108683–4113352	1032	13	114.98	9.06	6
*BraABCG30*	Bra037088	ABCG	A05	9949465–9955806	1395	24	157.63	8.7	11
*BraABCG31*	Bra038121	ABCG	A05	10351210–10356004	1054	13	117.35	8.74	6
*BraABCG32*	Bra030437	ABCG	A05	11199344–11202776	692	8	76.9	9.62	7
*BraABCG33*	Bra033878	ABCG	A05	14752718–14755091	641	5	71.73	8.73	6
*BraABCG34*	Bra031249	ABCG	A05	16677313–16681012	691	8	77.25	9.57	6
*BraABCG35*	Bra027171	ABCG	A05	19501072–19510210	1922	21	214.77	8.2	15
*BraABCG36*	Bra039669	ABCG	A06	615172–622258	1131	17	126.02	8.62	7
*BraABCG37*	Bra039668	ABCG	A06	623049–628079	1086	13	120.54	9.06	5
*BraABCG38*	Bra018911	ABCG	A06	1309663–1313997	630	9	70.25	9.69	5
*BraABCG39*	Bra026157	ABCG	A06	5834476–5845414	1462	10	164.84	8.18	13
*BraABCG40*	Bra026156	ABCG	A06	5847994–5853304	1444	9	162.85	7.87	13
*BraABCG41*	Bra026124	ABCG	A06	5958546–5964576	1413	19	159.5	7.26	13
*BraABCG42*	Bra025944	ABCG	A06	6859917–6863030	676	10	75.41	9.08	6
*BraABCG43*	Bra025424	ABCG	A06	21597809–21603945	1401	21	159	9.04	13
*BraABCG44*	Bra025152	ABCG	A06	23183495–23186962	495	5	54.41	9.81	0
*BraABCG45*	Bra011981	ABCG	A07	10786658–10789875	732	10	81.15	8.83	7
*BraABCG46*	Bra003137	ABCG	A07	11540111–11546317	1476	22	166.42	7.19	12
*BraABCG47*	Bra003208	ABCG	A07	11881016–11883287	707	2	78.84	8.58	7
*BraABCG48*	Bra003527	ABCG	A07	13638753–13643759	1440	7	162.09	8.14	13
*BraABCG49*	Bra003903	ABCG	A07	15648029–15654679	650	4	71.86	8.58	7
*BraABCG50*	Bra016095	ABCG	A07	19433991–19439446	661	4	72.22	8.94	7
*BraABCG51*	Bra030948	ABCG	A08	1007674–1009440	588	1	66.2	9.77	7
*BraABCG52*	Bra014326	ABCG	A08	1588811–1592015	737	11	80.54	5.52	3
*BraABCG53*	Bra016584	ABCG	A08	19155230–19158138	701	10	78.34	9.05	6
*BraABCG54*	Bra016669	ABCG	A08	19501741–19507488	1437	9	162.15	8.49	15
*BraABCG55*	Bra023202	ABCG	A09	20771853–20775484	727	6	80.93	9.04	7
*BraABCG56*	Bra007006	ABCG	A09	26775470–26782652	946	6	104.76	9.64	6
*BraABCG57*	Bra007141	ABCG	A09	27530807–27533042	708	2	78.87	8.05	6
*BraABCG58*	Bra002941	ABCG	A10	6596284–6598609	260	6	29.01	5.4	0
*BraABCG59*	Bra002234	ABCG	A10	10746710–10748152	480	1	54.6	6.9	6
*BraABCG60*	Bra008817	ABCG	A10	13968960–13971140	726	1	80.27	10.01	6
*BraABCG61*	Bra009203	ABCG	A10	15623186–15628005	755	9	83.41	9.75	5
*BraABCG62*	Bra035353	ABCG	Scaffold000104	110989–113136	715	1	79.84	7.54	6
*BraABCG63*	Bra039378	ABCG	Scaffold000164	191507–194585	681	9	77.46	8.59	5
*BraABCI1*	Bra011442	ABCI	A01	2152914–2154414	273	5	29.68	7.3	0
*BraABCI2*	Bra039560	ABCI	A01	11870454–11879989	1330	9	150.68	5.82	0
*BraABCI3*	Bra033786	ABCI	A01	14243301–14249351	298	4	32.71	8.9	0
*BraABCI4*	Bra034089	ABCI	A01	27521492–27524437	810	7	89.58	4.82	0
*BraABCI5*	Bra022481	ABCI	A02	9506899–9508775	329	10	35.97	8.46	0
*BraABCI6*	Bra034020	ABCI	A02	10133904–10156797	5408	60	611.88	5.02	0
*BraABCI7*	Bra033064	ABCI	A02	21580642–21581082	146	1	15.7	9.88	0
*BraABCI8*	Bra005714	ABCI	A03	252367–253876	301	6	34.45	10.34	0
*BraABCI9*	Bra005733	ABCI	A03	342459–347749	1104	8	123.03	7.18	0
*BraABCI10*	Bra001297	ABCI	A03	15733735–15737050	809	8	89.55	4.85	0
*BraABCI11*	Bra001373	ABCI	A03	16049519–16054747	970	13	107.23	6.1	0
*BraABCI12*	Bra025641	ABCI	A04	7327396–7328438	208	4	23.56	8.94	0
*BraABCI13*	Bra005049	ABCI	A05	3167954–3168847	297	1	33.65	9.46	6
*BraABCI14*	Bra035791	ABCI	A05	17228607–17232934	777	10	85.93	7.69	1
*BraABCI15*	Bra029859	ABCI	A05	21964512–21970206	950	13	105.22	5.54	0
*BraABCI16*	Bra029795	ABCI	A05	22242003–22245164	809	8	89.54	4.8	0
*BraABCI17*	Bra010169	ABCI	A06	20423304–20425052	477	2	52.95	5.89	0
*BraABCI18*	Bra003123	ABCI	A07	11453085–11457040	810	8	89.86	4.79	0
*BraABCI19*	Bra004271	ABCI	A07	17733376–17734681	264	2	28.83	5.03	0
*BraABCI20*	Bra010588	ABCI	A08	15346788–15355544	1939	9	217.72	4.96	0
*BraABCI21*	Bra030549	ABCI	A08	21373671–21375232	291	6	32.53	6.51	0
*BraABCI22*	Bra027807	ABCI	A09	6028957–6029646	229	1	25.86	10.52	0
*BraABCI23*	Bra027825	ABCI	A09	9029114–9029966	225	2	24.65	9.86	2
*BraABCI24*	Bra027530	ABCI	A09	13544344–13546011	277	6	30.88	7.58	0
*BraABCI25*	Bra029516	ABCI	A09	17422682–17424419	552	2	61.27	5.81	0
*BraABCI26*	Bra006978	ABCI	A09	26635532–26639138	811	8	89.98	4.91	0
*BraABCI27*	Bra026759	ABCI	A09	33595619–33596262	132	2	15.22	8.98	3
*BraABCI28*	Bra008779	ABCI	A10	13812509–13814463	274	10	30.39	9.21	0
*BraABCI29*	Bra009601	ABCI	A10	17396600–17398343	325	6	36.53	8.62	0
*BraABCI30*	Bra040852	ABCI	Scaffold000281	30428–32476	195	4	22.15	8.94	0

*MW, molecular weight; PI, isoelectric point; TMs, transmembrane helices number*.

Understanding the evolutionary relationship between BraABC proteins and AtABC proteins could contribute to classifying and assessing the potential functions of BraABCs. The NBD domains of the 162 BraABC proteins and 117 AtABC proteins (117 AtABC proteins contained the NBD domain) were aligned, and an unrooted phylogenetic tree was constructed by MEGA6 using the Maximum Likelihood (ML) method with 1,000 bootstraps. The AtABC proteins of each subfamily were grouped together, whereas the proteins of ABCI were dispersive in the phylogenetic tree. Overall, BraABC proteins followed the phylogenetic pattern of *Arabidopsis* (Supplementary Figure 1). Based on the phylogenetic relationship with AtABCs, BraABCs were also divided into eight subfamilies: ABCA, ABCB, ABCC, ABCD, ABCE, ABCF, ABCG and ABCI. ABCG, ABCB, ABCI, and ABCC were the largest subfamilies containing 63, 38, 30, and 21 members, respectively. ABCA, ABCE, and ABCF included 11, 7, and 7 members, respectively. Only 2 members were identified in ABCD (Table 1). The protein domain organizations of BraABCs in each subfamily were consistent with that of *Arabidopsis* (Verrier et al., 2008).

**Figure 1:**
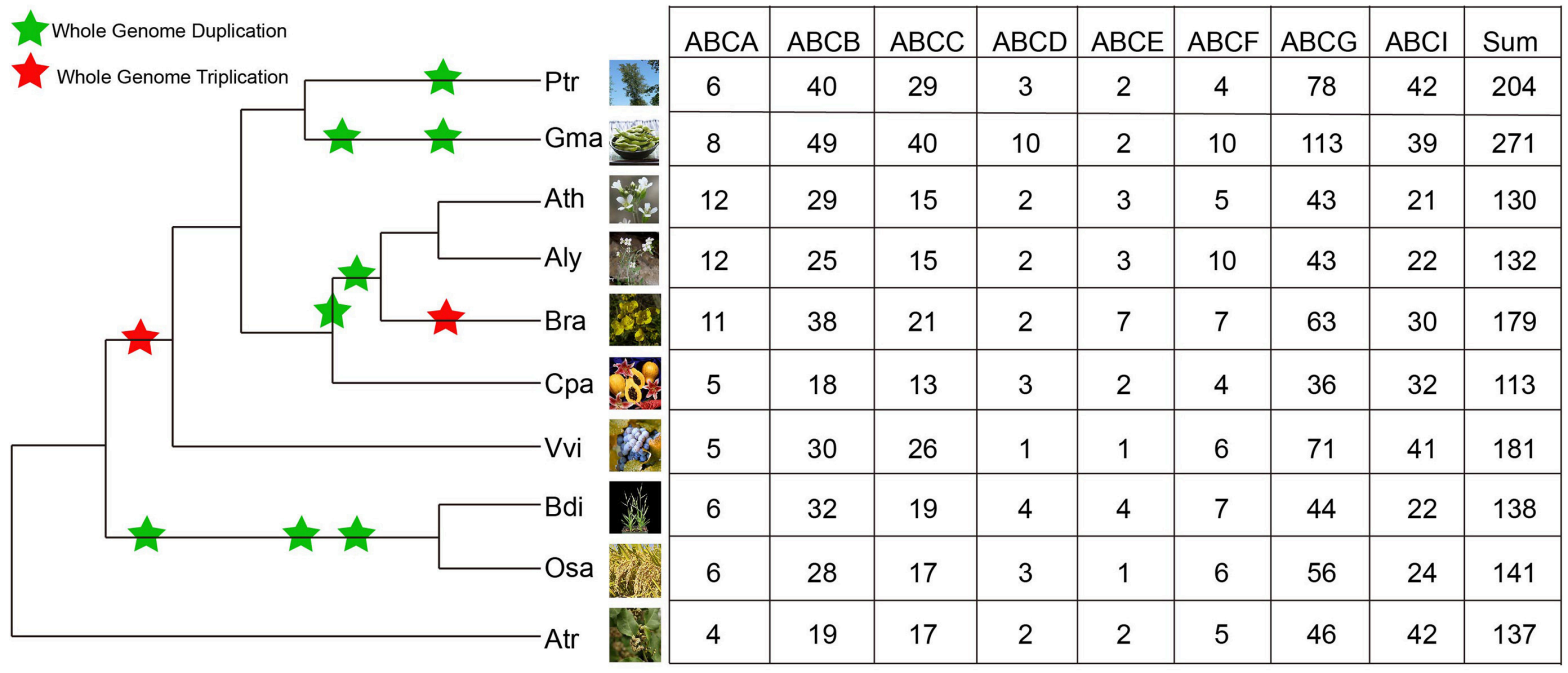
**Distribution of ABCs in different plant species and subfamilies**. Information regarding genome duplication or triplication was obtained from the Plant Genome Duplication Database. The phenotypic picture of each species was obtained from Phytozome 10. Ptr, Gma, Ath, Aly, Bra, Cpa, Vvi, Bdi, Osa, and Atr are the abbreviations of *P. trichocarpa, G. max, A. thaliana, A. lyrate, B. rapa, C. papaya, V. vinifera, B. distachyon, O. sativa*, and *A. trichopoda*, respectively.

Similarly, Hmmsearch searches were also conducted in the selected angiosperms: *P*. *trichocarpa, G. max, A. lyrate, C. papaya, V. vinifera, B. distachyon, O. sativa*, and *A. trichopoda*. The number of identified ABCs in these species ranged from 113 to 271 (Figure 1). There were 271 ABCs existing in the *G. max* genome, while the members in other species ranged from 113 in *C. papaya*, 130 in *Arabidopsis*, 132 in *A. lyrate*, 137 in *A. trichopoda*, 138 in *B. distachyon*, 141 in *O. sativa*, 179 in *B. rapa*, 181 in *V. vinifera* and 204 in *P. trichocarpa*. The identified ABCs in these species are listed in Supplementary Table 1.

**Figure 2:**
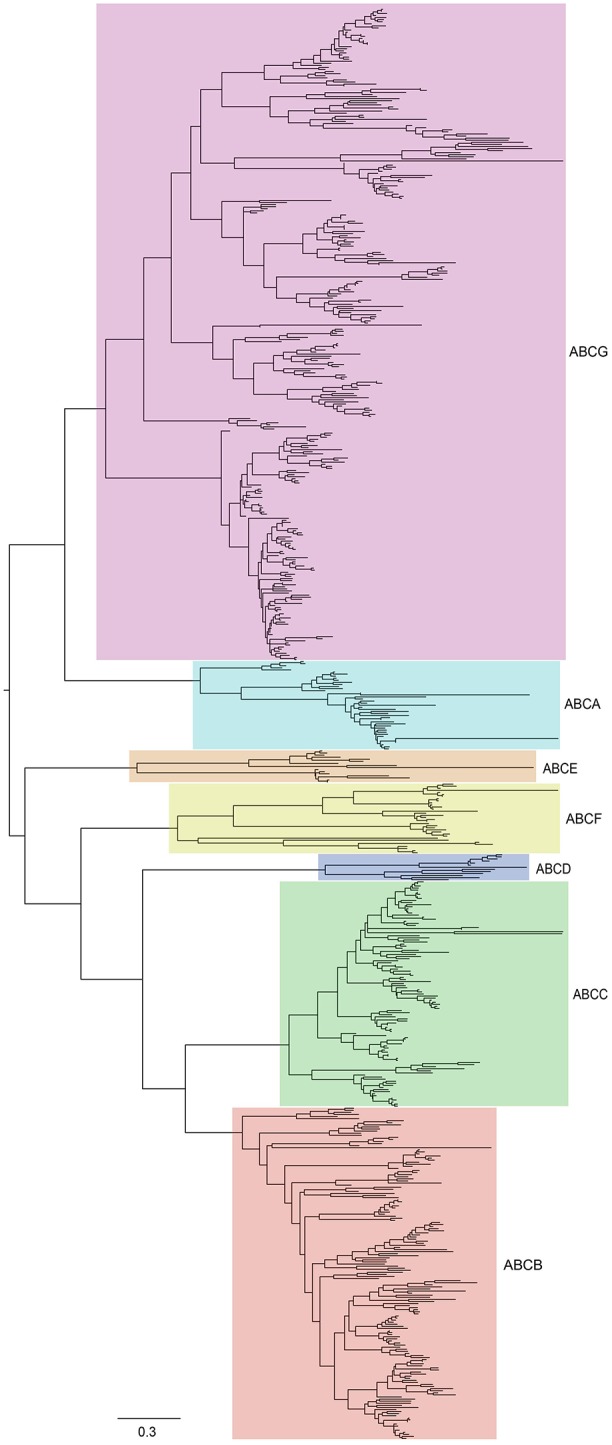
**Phylogenetic relationships among ABCs in six plant species, including ***Arabidopsis***, ***B. rapa***, ***C. papaya***, ***V. vinifera***, ***O. sativa***, and ***A. trichopoda*****. The unrooted tree was constructed by MEGA6 using the maximum-likelihood (ML) procedure with 1,000 bootstrap replicates. The tree can be divided into seven major clades (ABCA-ABCG). All subfamilies have been labeled by different colors.

To gain insight into the evolutionary history and relationship of ABCs in plants, we constructed a phylogenetic tree using ABCs of six representative species, including *Arabidopsis* (Verrier et al., 2008), *B. rapa, C. papaya, V. vinifera, O. sativa*, and *A. trichopoda*. In contrast with the ABCA-ABCG members, some ABCI proteins lacked the NBD domain, and some ABCI members were scattered in the phylogenetic tree, although they contained the NBD domain. Therefore, our phylogenetic tree was constructed using the NBD domains of ABCA-ABCG members from these species. As shown in Figure 2, seven distinct clades were clearly distinguished based on the tree topology, namely ABCA, ABCB, ABCC, ABCD, ABCE, ABCF, and ABCG, suggesting that these groups have diverged prior to the divergence of angiosperm. Next, the copy number of ABCs in each subfamily of these species was quantified (Figure 1). In each studied species, the copy numbers of different subfamilies varied greatly. For the copy numbers of subfamilies in all of the studied species, we found that ABCB, ABCC, ABCG, and ABCI were the larger subfamilies. ABCA, ABCD, ABCE, and ABCF contained a small number of members, and the copy numbers of ABCD, ABCE, and ABCF were relatively constant. *B. rapa* has undergone a further WGT since its divergence from *Arabidopsis*. Compared with *Arabidopsis*, the expansion of the ABC gene family in *B. rapa* was largely the result of the expansion of ABCs in ABCB, ABCC, ABCG, and ABCI (Figure 1).

The 179 BraABCs were designated as *BraABCA1* through *BraABCI30* according to their respective subfamily and their location on the chromosome (Table 1). The comprehensive information of BraABCs, including the locus name, gene location, predicted protein length, exon number, molecular weight (MW), isoelectric point (PI) and transmembrane helices number (TMs), is listed in Table 1. The size of the BraABC proteins varied from 118 (*BraABCB31*) to 5,408 (*BraABCI6*) amino acids, with an average of ~984 amino acids. There were few differences in the protein lengths of ABCC, ABCE and ABCF, whereas the protein lengths of ABCB, ABCG and ABCI changed greatly. The MWs of the BraABC proteins ranged from 12.87 kDa (*BraABCB31*) to 611.88 kDa (*BraABCI6*). The variation range of PI was from 4.79 (*BraABCI18*) to 10.52 (*BraABCI22)*, indicating that these BraABC proteins might exist and function in different parts of the cells. Most of the BraABC proteins (162 out of 179, 90.5%) presented multiple exons and sixty proteins (33.5%) that contained more than 10 exons. The majority of the BraABC proteins contained transmembrane helices (TMs), except for all of the ABCE and ABCF proteins as well as parts of the ABCI proteins. The ubiquity of TMs was in accordance with the transmembrane transport functions of ABCs. Pairwise comparisons of the full-length BraABC protein sequences within each subfamily were conducted. The protein sequences within ABCA, ABCB, and ABCI exhibited higher identities than other subfamilies (Supplementary Figure 2). In contrast, the sequence identities of ABCD and ABCG were lower, indicating that the degree of sequence divergence of the two subfamilies was higher than other subfamilies. The other families, such as ABCC, ABCE, and ABCF exhibited medium sequence identities.

### Chromosomal locations and tandem array of BraABCs

**Figure 3:**
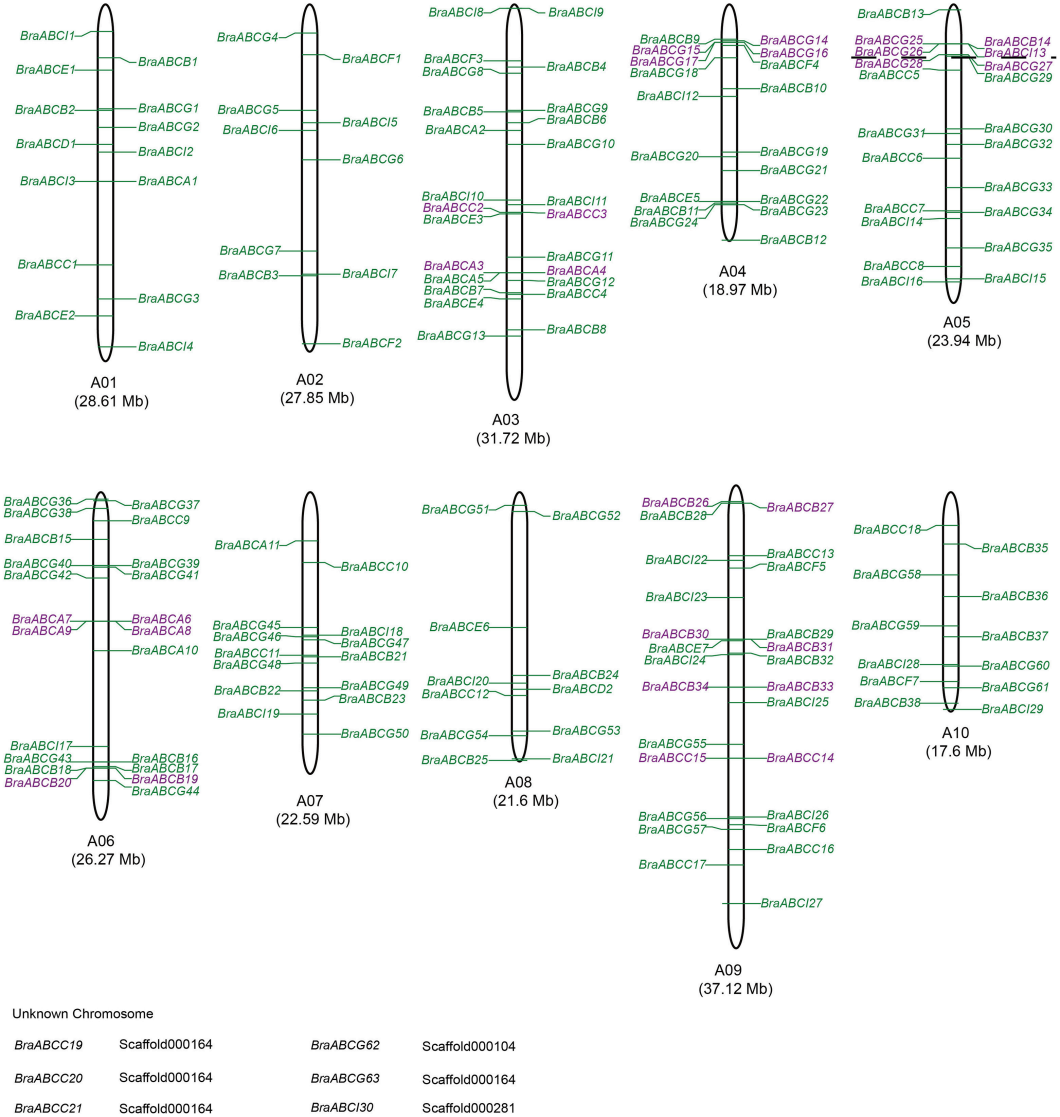
**Chromosomal locations of BraABCs**. Chromosome numbers and lengths are represented at the bottom of each chromosome. Tandem arrays of BraABCs are marked in purple. Six BraABCs could not be anchored onto a specific chromosome.

A total of 173 BraABCs were located on the ten chromosomes (96.7%), and the other 6 genes were located on unanchored scaffolds (Figure 3). The BraABCs were unevenly distributed on the ten chromosomes. The largest number of BraABCs was found on chromosome A09 (26 genes), followed by A03 (25 genes). Chromosomes A06 and A05 also contained more than 20 BraABCs (22 and 21, respectively). In contrast, chromosomes A04, A01, A07, A10, A08, and A02 contained 18, 14, 14, 12, 11, and 10 BraABCs, respectively. Our results indicated that BraABCs within each subfamily were also unevenly distributed on the ten chromosomes.

Simultaneously, we further identified tandem arrays of BraABCs along the 10 *B. rapa* chromosomes. A tandem array was defined as multiple members of BraABCs occurring within the same or neighboring intergenic regions. As shown in Figure 3, 11 BraABCs clusters (genes marked in purple) containing 28 tandemly duplicated genes were identified on chromosomes A03, A04, A05, A06, and A09.

### Motif composition and exon-intron organization of BraABC proteins

To better understand the conservation and diversity of motif compositions and gene structures of BraABCs, the conserved motifs and exon-intron organization of BraABCs were analyzed.

**Figure 4:**
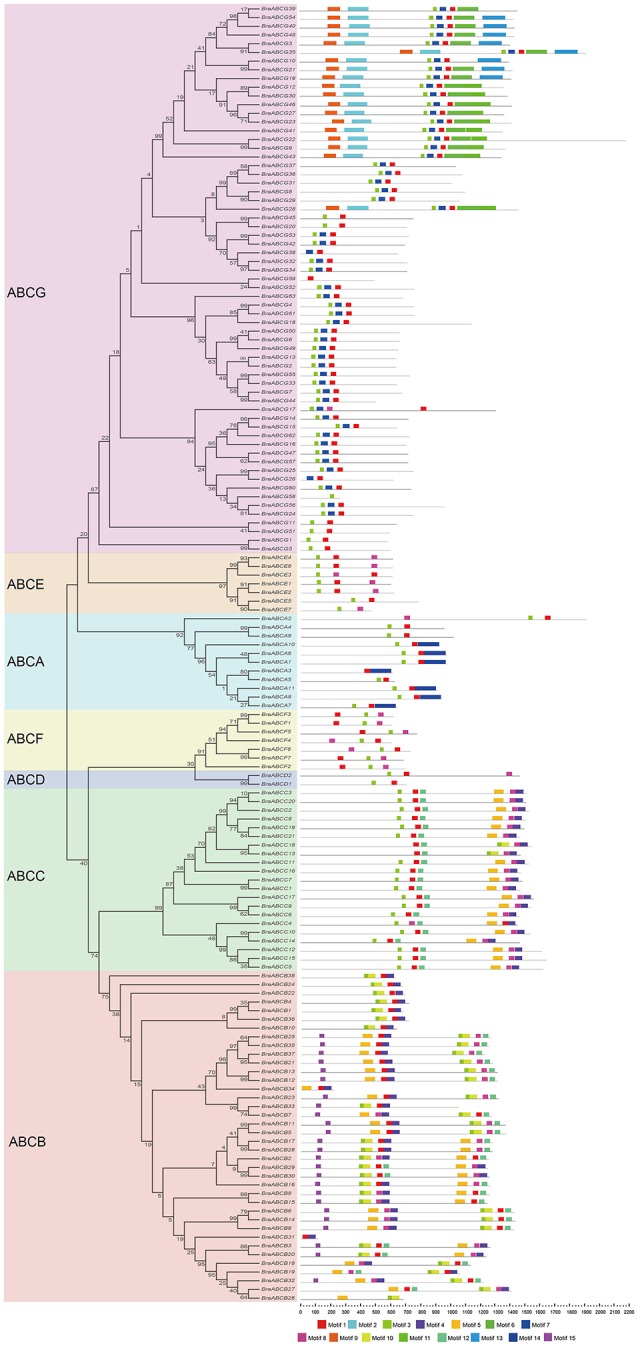
**Conserved motifs of BraABCs according to the evolutionary relationship**. The conserved motifs were elucidated using MEME with complete protein sequences. Different motifs are represented by different colors, numbered 1–15 at the bottom. The black lines represent the non-conserved sequences.

The conserved motifs of BraABC proteins in ABCA-ABCG were analyzed using MEME software, and we identified 15 conserved motifs (Figure 4). The lengths of the conserved motifs were between 26 and 154 amino acids. The number of the conserved motifs in each BraABC protein varied from 1 to 8. Several motifs were widely spread among BraABC proteins; for instance, motifs 1 and 3 were present in the most proteins of ABCA-ABCG. In contrast, other motifs were specific to only one or two subfamilies. For example, motifs 4, 5, 8, and 12 were specific to ABCB and ABCC, motifs 10 and 15 were specific to ABCB, and motif 14 was specific to ABCA. ABCG contained the specific motifs 2, 6, 7, 9, 11, and 13, and these motifs were probably required for specific protein functions. The varieties of motif compositions in different subfamilies suggested sources of functional differentiation in BraABCs during the evolutionary process. BraABC proteins grouped into the same subfamily exhibited similar motif characteristics, suggesting functional similarities for members in the same subfamily. Logos of the 15 identified motifs are shown in Supplementary Figure 3.

We also analyzed the exon-intron organizations of BraABCs using GSDS 2.0. Similar to the motif composition, most of the BraABCs within the same subfamily generally exhibited similar gene structure in terms of the numbers and lengths of introns and exons. Moreover, BraABCs that had a closely phylogenetic relationship and shared a more similar gene structure (Supplementary Figure 4).

### Conservation of BraABCs following the whole genome triplication event

**Figure 5:**
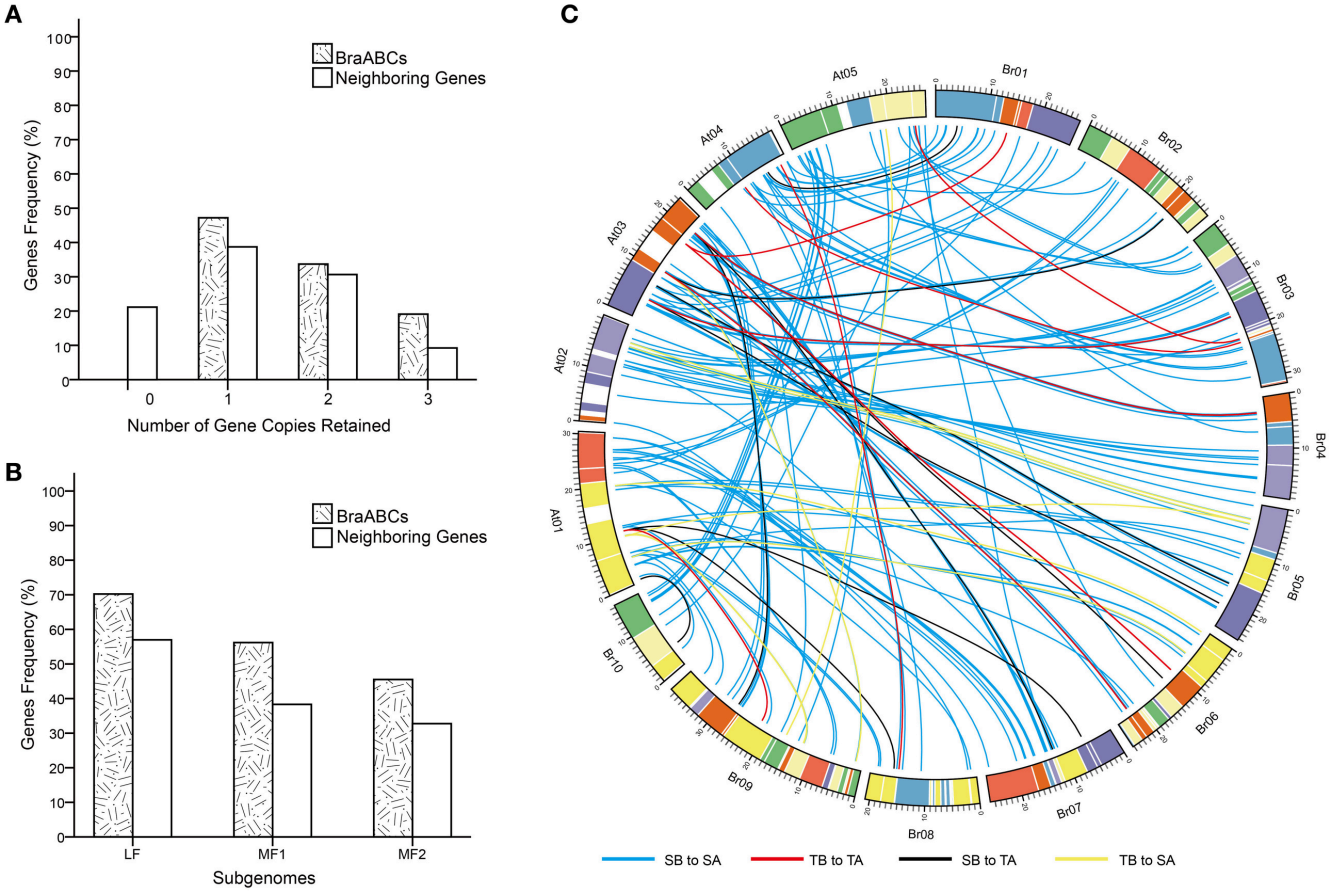
**Retention of BraABCs and their neighboring genes (10 flanking genes on either side of BraABCs) in ***B. rapa***. (A)** Retention by the number of homologous copies in the syntenic region. **(B)** Retention of homologs among the three subgenomes of *B. rapa*. **(C)** Collinear correlations of ABCs between *Arabidopsis* and *B. rapa*. The *B. rapa* and *Arabidopsis* chromosomes are colored according to the inferred ancestral chromosomes following an established convention. SA, SB, TA, and TB are the abbreviations of a single copy of *Arabidopsis*, a single copy of BraABC, a tandem array of *Arabidopsis* and a tandem array of BraABCs, respectively. The four collinear correlations of SB to SA, TB to TA, SB to TA, and TB to SA are represented by blue, red, black, and yellow lines, respectively.

To explore the influences of WGT on the evolution of BraABCs, we studied the conservation of BraABCs after WGT. After diverging from *Arabidopsis*, the gene number of the *B. rapa* genome was ~42,000, which is considerably lower than the simple triplication of the ~30,000 genes in the *Arabidopsis* genome. Therefore, substantial genes must have been lost (fractionation) in *B. rapa* after triploidization (Wang et al., 2011). To evaluate the fractionation extent of BraABCs, we compared the retention of BraABCs relative to the set of 3,580 neighboring genes (10 on either side), flanking the 179 BraABCs (Supplementary Table 2). In comparison, 47.2% of BraABCs were retained in one copy, which was greater than the retention of neighboring genes (38.7%). A similar percent (33.7 and 30.6%, respectively) of BraABCs and neighboring genes were retained in the two copies. And more BraABCs (19.1%) were retained in the three copies than their neighboring genes (9.2%) (Figure 5A).

The triplicated *B. rapa* genome can be divided into three subgenomes: LF, MF1, and MF2. The subgenomes were grouped based on the level of gene loss relative to *Arabidopsis* since the WGT 13–17 MYA. Compared with their neighboring genes, more BraABCs were significantly retained in all three subgenomes. Consistent with the whole genome level, BraABCs distributed in the LF subgenome were more than two other subgenomes (Figure 5B). Given the well-conserved synteny between *Arabidopsis* and *B. rapa* (Cheng et al., 2012), in our study, there were four collinear relations for ABCs between *B. rapa* and *Arabidopsis*: a single copy of BraABC to a single copy of *Arabidopsis* (SB to SA); a tandem array of BraABCs to a tandem array of *Arabidopsis* (TB to TA); a single copy of BraABC to a tandem array of *Arabidopsis* (SB to TA); and a tandem array of BraABCs to a single copy of *Arabidopsis* (TB to SA). The four syntenic relations were presented using the Circos program (Figure 5C). On the whole, BraABCs were preferentially retained, consistent with the gene balance hypothesis and demonstrating the significance of ABCs in *B. rapa*.

### *Cis*-elements of BraABCs involved in transcriptional regulation

To identify *cis*-elements related to transcriptional regulation, 2 kb promoter regions for each of the 179 BraABCs were identified. Gaps existed in the promoter regions of 13 BraABCs after assembling the *B. rapa* genome. Therefore, these BraABCs were excluded in the following study. Next, we applied the Plant *Cis*-acting Regulatory DNA Elements (PLACE) website to analyze the *cis*-elements and identified a total of 291 different *cis*-elements in all of the studied BraABCs.

A total of 13 common *cis*-regulatory elements were identified in all of the promoter regions of the BraABCs, which were highly conserved among all of the studied BraABCs (Table 2). Two common *cis*-regulatory elements, WRKY71OS and GT1CONSENSUS were responsive to plant hormones, including ABA, GA, and SA, suggesting that ABA, GA, and SA could affect the expression levels of BraABCs. Some common *cis*-regulatory elements were responsive to stresses, such as pathogens (WRKY71OS) and wounds (WBOXNTERF3). Of the 13 common *cis*-regulatory elements, GATABOX, IBOXCORE, and GT1CONSENSUS were required for transcriptional regulation by light. DOFCOREZM participated in carbon metabolism, and GATABOX played a role in molecular light switching; therefore, we speculated that BraABCs were likely to participate in energy metabolism. Two common *cis*-elements, POLLEN1LELAT52 and GTGANTG10, were required for transcriptional regulation in pollen, suggesting that BraABCs might be involved in the reproductive process.

**Table 2:** **Common putative ***cis***-elements identified in the promoter sequences of BraABCs**.

***Cis*-element**	**Signal sequence**	**SITE**	**Expression pattern**
CAATBOX1	CAAT	S000028	Seed
ARR1AT	NGATT	S000454	Response regulator
WRKY71OS	TGAC	S000447	GA, ABA, PR proteins, plant defenses
DOFCOREZM	AAAG	S000265	Leaf, shoot, carbon metabolism
WBOXNTERF3	TGACY	S000457	Wound
NODCON2GM	CTCTT	S000462	Nodule
GATABOX	GATA	S000039	Leaf, shoot, light, molecular light switches
IBOXCORE	GATAA	S000199	Leaf, shoot, light regulation
POLLEN1LELAT52	AGAAA	S000245	Pollen
CACTFTPPCA1	YACT	S000449	C4 plant, mesophyll
TAAAGSTKST1	TAAAG	S000387	Guard cell, K+ influx channel
GT1CONSENSUS	GRWAAW	S000198	Leaf, shoot, light, SA
GTGANTG10	GTGA	S000378	Pollen

We also identified unique *cis*-element sequences that are present in the promoter regions of unique BraABCs. In total, 40 unique *cis*-elements were identified in the promoter regions of the 36 BraABCs (Supplementary Table 3). Interestingly, 3 unique *cis*-elements (ACGTSEED3, ABREMOTIFIOSRAB16B, and VOZATVPP) were presented in only *BraABCF3*. *BraABCG18* and *BraABCB10* both contained two unique *cis*-elements. There was only one unique *cis*-element present in the other 33 BraABCs. The unique *cis*-element of these genes indicated their expression specificity.

### Gene expression of BraABCs in different tissues

**Figure 6:**
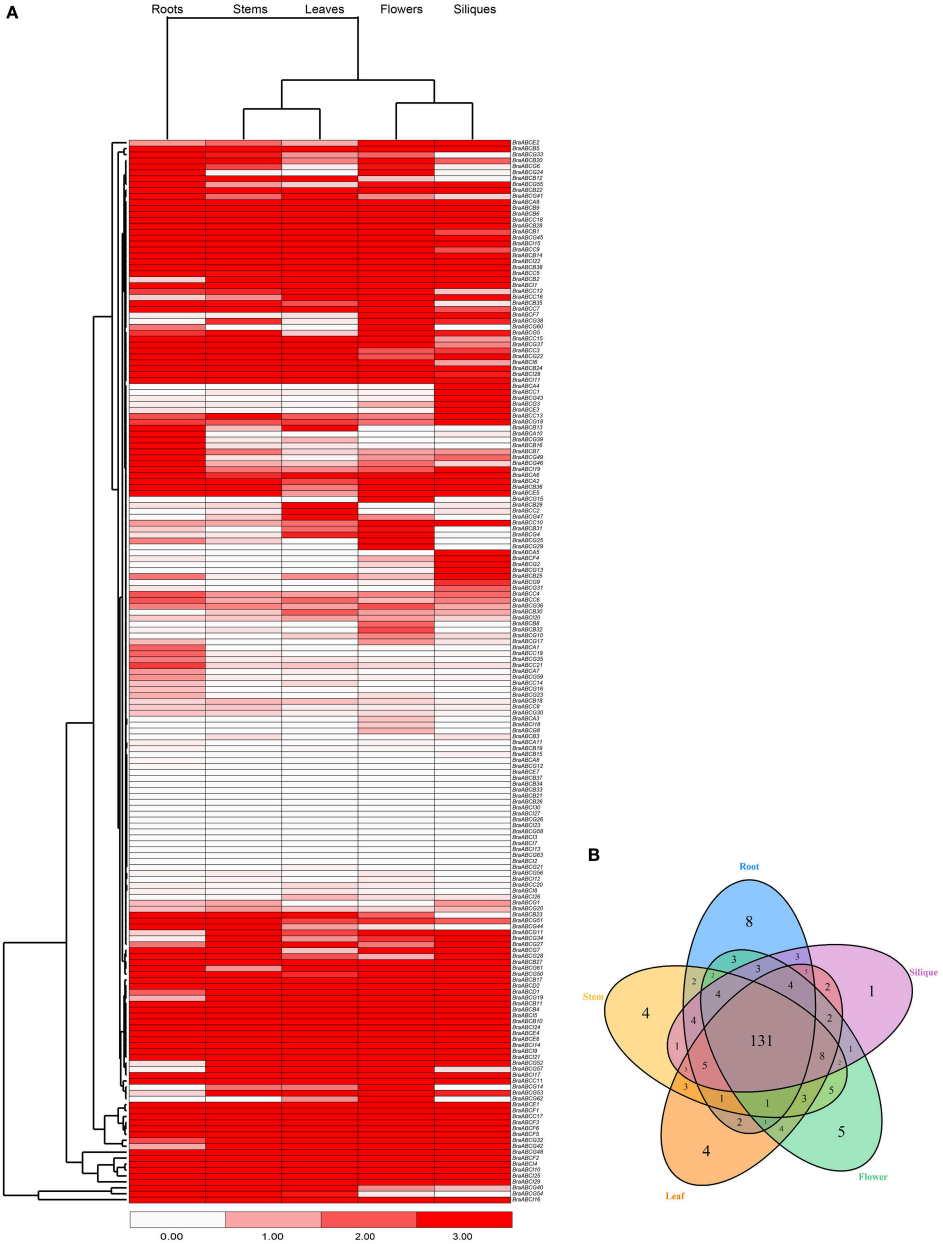
**Expression patterns of BraABCs in five tissues. (A)** Heat map of expression profiles (in log_2_-based FPKM) for BraABCs in the five tissues of roots, stems, leaves, flowers, and siliques. The expression levels are represented by the color bar. **(B)** Venn diagram analysis of the tissue-expression of BraABCs.

To characterize the expression patterns of individual BraABCs in different tissues, we used publicly available RNA-Seq data of different tissues as a resource (Tong et al., 2013). A heat map displaying the expression profiles of BraABCs in roots, stems, leaves, flowers and siliques was generated, and the BraABCs were clustered by their expression patterns. As shown in Figure 6A, the majority of BraABCs presented different expression patterns, whereas a few of them exhibited similar expression patterns. A total of 165 BraABCs (92.2%) were determined as being expressed in at least one tissue, and 117 BraABCs (65.4%) expressed in all tissues, including 3 ABCA genes, 26 ABCB genes, 19 ABCC genes, 2 ABCD genes, 5 ABCE genes, 6 ABCF genes, 37 ABCG genes and 19 ABCI genes. Relatively, the expression of 14 BraABCs was not detected in any of the five tissues. Some BraABCs also exhibited tissue-specific expression, and there were 8, 4, 4, 5, and 1 tissue-specific BraABCs in the roots, stems, leaves, flowers and siliques, respectively (Figure 6B), suggesting that these genes may play specific roles in the relevant tissues. As shown in Figure 6A, some genes of ABCA, ABCB, and ABCG exhibited variable expression profiles throughout the five tissues, such as *BraABCA10, BraABCB13*, and *BraABCG25*. In contrast, the majority of genes in ABCC, ABCD, ABCE, ABCF, and ABCI presented relatively consistent expression levels across the five tissues, such as *BraABCC18, BraABCD2, BraABCE6, BraABCF1*, and *BraABCI14*. As shown in Figure 6A, we found the expression profiles of BraABCs in the roots exhibited large differences compared with the other four tissues, whereas similar expression patterns of BraABCs were observed for the stems and leaves, and the flowers and siliques.

### Syntenic BraABC paralog pairs among the three subgenomes of *B. rapa*

The three subgenomes of *B. rapa* are the evolutionary products of WGT and a lot of synteny blocks among them. Syntenic paralogs are genes that are located in the syntenic fragments. Syntenic BraABC paralog pairs among LF, MF1, and MF2 were identified by searching “syntenic gene” in the BRAD (Cheng et al., 2011). In total, 76 pairs of BraABC syntenic paralogs were identified among the three subgenomes (Table 3). Synonymous (*Ks*) and non-synonymous (*Ka*) values were calculated to explore the selective pressures on these paralog pairs. We found that the ω (= *Ka*/*Ks*) ratios of 10 syntenic paralogs (13.16%) were more than 1, indicating positive selection on these BraABC paralogs. The *ABCC21_ABCC3* pair had the highest ω ratios, with a ratio of 1.9264. The rest of the paralogs were under purifying selection, with ω ratios lower than 1. Among them, the ω ratios of 22 paralog pairs were lower than 0.1, suggesting that these gene pairs underwent strong purifying selection stress, making the functions of these paralog pairs trend toward relative similarity. Furthermore, the duplication time of these paralogs pairs were estimated by using a relative *Ks* measure as a proxy for time. The estimated duplication time of BraABC paralog pairs indicated that it spanned from 3.45 to 42.2 MYA, with an average duplication time of ~15.13 MYA, which was consistent with the recent WGT time of *B. rapa* (13–17 MYA) (Wang et al., 2011).

**Table 3:** *****Ka/Ks*** calculation of each pair of syntenic BraABC paralogs among three subgenomes**.

**Syntenic paralog pairs**	**S-Sites**	**N-Sites**	***Ka***	***Ks***	***Ka/Ks***	**Selection pressure**	**Duplication time (MYA)**
*ABCI7_ABCB28*	101.7	312.3	0.0361	0.2692	0.1340	Purify selection	8.97
*ABCA7_ABCA1*	426.3	1430.7	0.1214	0.6921	0.1754	Purify selection	23.07
*ABCA7_ABCA10*	387.3	1325.7	0.2713	1.1081	0.2449	Purify selection	36.94
*ABCA8_ABCA1*	637.9	2110.1	0.1567	0.6438	0.2433	Purify selection	21.46
*ABCG55_ABCG33*	459.5	1457.5	0.0181	0.3379	0.0534	Purify selection	11.26
*ABCA8_ABCA10*	548.6	1884.4	0.3411	0.7180	0.4751	Purify selection	23.93
*ABCI3_ABCA10*	184.7	634.3	0.2605	0.9483	0.2747	Purify selection	31.61
*ABCB36_ABCB4*	513.5	1622.5	0.0358	0.3744	0.0955	Purify selection	12.48
*ABCC21_ABCC3*	833.1	2877.9	0.4073	0.2114	1.9264	Positive selection	7.05
*ABCC8_ABCC3*	907.0	2972.0	0.3718	0.2640	1.4083	Positive selection	8.80
*ABCG39_ABCG54*	998.5	3303.5	0.0407	0.4111	0.0991	Purify selection	13.70
*ABCC14_ABCC12*	828.4	2879.6	0.4574	0.3221	1.4202	Positive selection	10.74
*ABCC14_ABCC10*	934.3	2995.7	0.0773	0.4210	0.1836	Purify selection	14.03
*ABCB18_ABCB27*	795.6	2522.4	0.0923	0.5602	0.1647	Purify selection	18.67
*ABCC20_ABCC3*	1, 022.8	3318.2	0.0711	0.3866	0.1838	Purify selection	12.89
*ABCB13_ABCB12*	918.0	2913.0	0.0214	0.3618	0.0592	Purify selection	12.06
*ABCG21_ABCG10*	998.9	3207.1	0.0312	0.3336	0.0935	Purify selection	11.12
*ABCG44_ABCG7*	357.6	1103.5	0.0806	0.2724	0.2959	Purify selection	9.08
*ABCC20_ABCC2*	948.6	3050.4	0.1483	0.1105	1.3417	Positive selection	3.68
*ABCC8_ABCC2*	972.1	3176.9	0.2673	0.7469	0.3578	Purify selection	24.90
*ABCC21_ABCC2*	946.1	3055.9	0.3096	0.6745	0.4590	Purify selection	22.48
*ABCB35_ABCB25*	854.1	2598.9	0.1366	0.1034	1.3209	Positive selection	3.45
*ABCC15_ABCC10*	935.2	3144.8	0.3639	0.5253	0.6928	Purify selection	17.51
*ABCC12_ABCC10*	969.0	3225.0	0.2990	0.8343	0.3583	Purify selection	27.81
*ABCG6_ABCG49*	482.0	1453.0	0.0674	0.4778	0.1411	Purify selection	15.93
*ABCA8_ABCI3*	190.6	658.4	0.1729	0.4297	0.4024	Purify selection	14.32
*ABCE1_ABCE4*	403.6	1336.4	0.0159	0.3465	0.0458	Purify selection	11.55
*ABCE1_ABCE6*	403.8	1336.2	0.0128	0.3867	0.0332	Purify selection	12.89
*ABCG42_ABCG53*	477.8	1541.2	0.0167	0.3074	0.0544	Purify selection	10.25
*ABCG14_ABCG47*	503.7	1587.3	0.1408	0.8319	0.1693	Purify selection	27.73
*ABCA1_ABCA10*	556.0	1850.0	0.3796	0.3834	0.9900	Purify selection	12.78
*ABCG35_ABCG3*	974.6	3264.4	0.0445	0.3155	0.1410	Purify selection	10.52
*ABCA7_ABCI3*	187.0	650.0	0.2065	0.4738	0.4358	Purify selection	15.79
*ABCC15_ABCC12*	1, 120.0	3644.0	0.0265	0.3014	0.0880	Purify selection	10.05
*ABCC8_ABCC20*	904.9	3043.1	0.3938	0.2233	1.7637	Positive selection	7.44
*ABCB11_ABCB5*	958.6	3031.4	0.0232	0.4174	0.0555	Purify selection	13.91
*ABCC8_ABCC21*	1, 000.5	3268.5	0.1138	0.6474	0.1758	Purify selection	21.58
*ABCB17_ABCB28*	904.7	2851.3	0.0149	0.4701	0.0316	Purify selection	15.67
*ABCB14_ABCB6*	1, 006.1	3190.9	0.0231	0.3068	0.0754	Purify selection	10.23
*ABCI15_ABCI11*	638.6	2133.4	0.0367	0.3204	0.1147	Purify selection	10.68
*ABCI16_ABCI4*	556.1	1861.9	0.0032	0.2802	0.0115	Purify selection	9.34
*ABCI16_ABCI10*	557.1	1864.0	0.0059	0.3252	0.0182	Purify selection	10.84
*ABCG2_ABCG13*	455.1	1455.9	0.0457	0.2647	0.1725	Purify selection	8.82
*ABCE4_ABCE6*	419.3	1395.7	0.0072	0.3489	0.0206	Purify selection	11.63
*ABCI4_ABCI10*	558.7	1865.3	0.0032	0.2760	0.0117	Purify selection	9.20
*ABCG16_ABCG47*	506.8	1587.2	0.0779	0.3979	0.1959	Purify selection	13.26
*ABCG15_ABCG47*	324.9	1016.1	0.2803	0.1506	1.8615	Positive selection	5.02
*ABCG17_ABCG47*	420.4	1373.6	0.3868	0.2192	1.7644	Positive selection	7.31
*ABCA9_ABCI3*	170.8	552.2	0.4139	0.5472	0.7564	Purify selection	18.24
*ABCB17_ABCI7*	101.8	312.2	0.0361	0.2976	0.1213	Purify selection	9.92
*ABCA6_ABCI3*	190.8	649.2	0.1545	0.6303	0.2452	Purify selection	21.01
*ABCI29_ABCI8*	203.3	675.7	0.0279	0.3872	0.0720	Purify selection	12.91
*ABCF6_ABCF4*	405.4	1346.6	0.0617	0.4685	0.1317	Purify selection	15.62
*ABCG41_ABCI27*	86.6	309.4	0.0364	0.4439	0.0821	Purify selection	14.80
*ABCA6_ABCA1*	656.7	2, 169.3	0.0777	0.3726	0.2086	Purify selection	12.42
*ABCA9_ABCA1*	459.7	1, 547.3	0.5582	0.9212	0.6060	Purify selection	30.71
*ABCA6_ABCA10*	585.3	1, 934.7	0.2449	1.1391	0.2149	Purify selection	37.97
*ABCA9_ABCA10*	441.0	1, 446.0	0.6087	0.6633	0.9177	Purify selection	22.11
*ABCG57_ABCG16*	506.5	1, 581.6	0.0697	0.3955	0.1764	Purify selection	13.18
*ABCG57_ABCG15*	350.3	1, 068.7	0.1476	0.6815	0.2165	Purify selection	22.72
*ABCG57_ABCG17*	467.6	1, 476.4	0.2053	1.2659	0.1622	Purify selection	42.20
*ABCC7_ABCC1*	918.1	2, 984.9	0.1512	0.1284	1.1774	Positive selection	4.280
*ABCB18_ABCB26*	436.6	1, 384.4	0.2332	0.1448	1.6106	Positive selection	4.83
*ABCG22_ABCG9*	983.5	3, 297.5	0.0375	0.4045	0.0926	Purify selection	13.48
*ABCB2_ABCB8*	890.4	2, 802.6	0.0566	0.3556	0.1592	Purify selection	11.85
*ABCG28_ABCG23*	972.9	3, 257.1	0.0592	0.4121	0.1437	Purify selection	13.74
*ABCG27_ABCG23*	977.1	3, 279.9	0.0649	0.3697	0.1755	Purify selection	12.32
*ABCE2_ABCE3*	416.3	1, 392.7	0.0879	0.6704	0.1311	Purify selection	22.35
*ABCG50_ABCG49*	488.3	1, 455.7	0.0566	0.4861	0.1164	Purify selection	16.20
*ABCG45_ABCG20*	485.2	1, 554.8	0.0363	0.4193	0.0867	Purify selection	13.98
*ABCG57_ABCG47*	513.3	1, 598.7	0.0484	0.4063	0.1192	Purify selection	13.54
*ABCG40_ABCG54*	997.6	3, 304.4	0.0432	0.4119	0.1050	Purify selection	13.73
*ABCG50_ABCG6*	493.9	1, 474.1	0.0617	0.5136	0.1202	Purify selection	17.12
*ABCG61_ABCG4*	550.0	1, 697.0	0.0279	0.3461	0.0806	Purify selection	11.54
*ABCG57_ABCG14*	480.0	1, 551.0	0.2200	0.3336	0.6596	Purify selection	11.12
*ABCI26_ABCI18*	554.5	1, 872.5	0.0322	0.3497	0.0920	Purify selection	11.66

*S-Sites, number of synonymous sites; N-Sites, number of non-synonymous sites; Ka, non-synonymous substitution rate; Ks, synonymous substitution; MYA, million years ago*.

### Evolutionary fates prediction of syntenic BraABC paralog pairs

**Figure 7:**
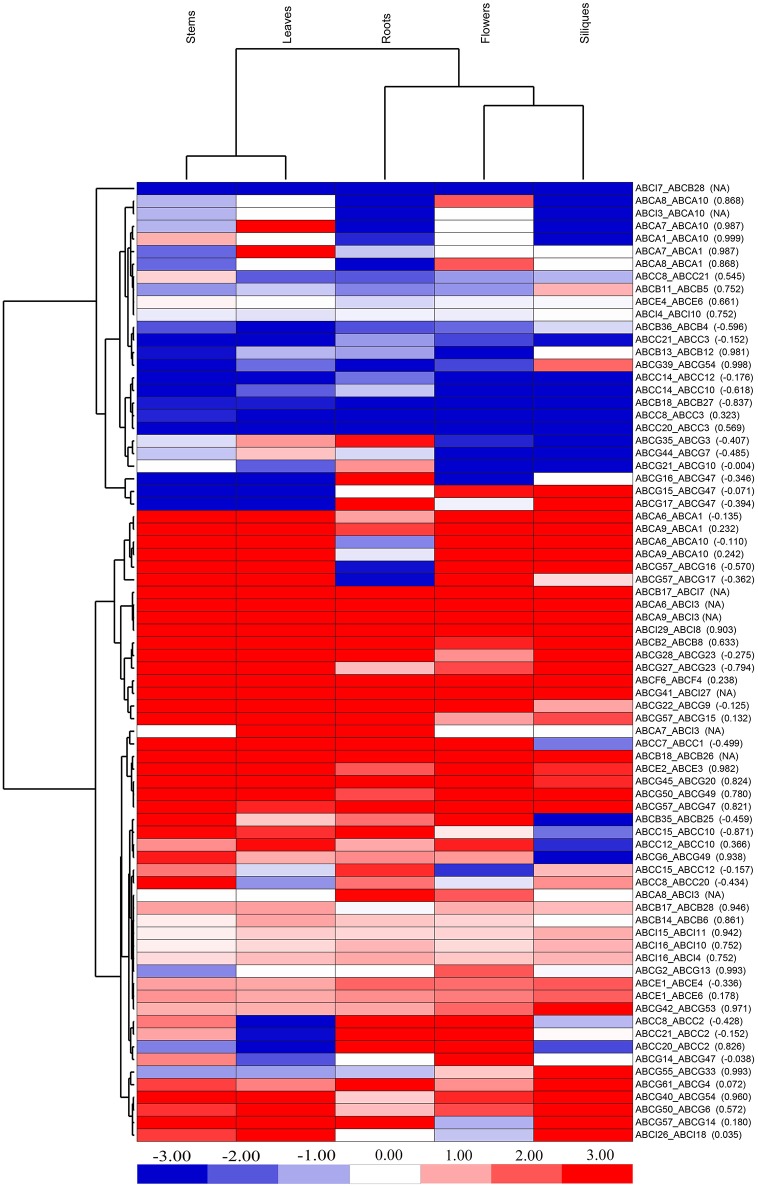
**Expression profiles of syntenic BraABC paralog pairs in the five tissues of roots, stems, leaves, flowers, and siliques**. The color bar on the bottom represents the log_2_-based ratio of FPKM values of the two genes in each paralog pair. The values in the bracket are Pearson correlation coefficients (PCCs) of paralog pairs measured by their expressions. NA stands for no available results for PCCs.

Profiling gene expression may provide an ample amount of information about the mode and tempo of duplicated genes. To predict the evolutionary fates of syntenic BraABC paralog pairs, we took advantage of the deep RNA-Seq data of *B. rapa* to investigate their expression profiles in different tissues, including roots, stems, leaves, flowers and siliques (Tong et al., 2013). Generally, if a Pearson correlation coefficient (PCC) of the two factors is greater than 0.6, we can infer that they have a close positive correlation. Among the 76 pairs of paralogs, 27 pairs had positive expression correlations across the five tissues, which were greater than 0.6 measured by the PCCs (Figure 7). Such high positive correlations among these paralog pairs probably indicated functional conservation or sub-functionalization for these paralog pairs after duplication. Simultaneously, 40 pairs of syntenic paralogs were negatively correlated with negative PCC values or exhibited little correlation with small positive PCC values in gene expression, suggesting neo-functionalization for these paralog pairs. Furthermore, there were 9 pairs of paralogs in which one copy of each pair did not express in any of the 5 tissues. Therefore, there were no available (NA) results of PCCs for these paralog pairs. Among the 9 paralog pairs, 8 pairs were composed of genes from two different subfamilies, and one copy of each of these paralog pairs was the ABCI gene. Interestingly, all of these ABCI genes were not expressed in any of the studied tissues, indicating that they probably became pseudogenes. Therefore, pseudogenization of these 9 pairs of paralogs may occur during evolution.

From the cluster of different tissues, the leaves clustered with the stems and the flowers clustered with the siliques in the heat map of BraABC paralog pairs indicated a similar expression profile (Figure 7). Taking an overall view of the cluster for BraABC paralog pairs, paralog pairs with similar evolutionary fates tended to further cluster together, confirming that the expression patterns of paralog pairs were related to their evolutionary fates after duplication. Approximately 42.1% (32 out of 76) of the paralog pairs presented no less than a two-fold expression difference (absolute value of log_2_ FPKM ratio ≥ 1) in all of the studied tissues. In brief, the expression correlation analysis of syntenic paralog pairs showed their functional roles in functional conservation, sub-functionalization, neo-functionalization and pseudogenization in the BraABC gene family.

**Figure 8:**
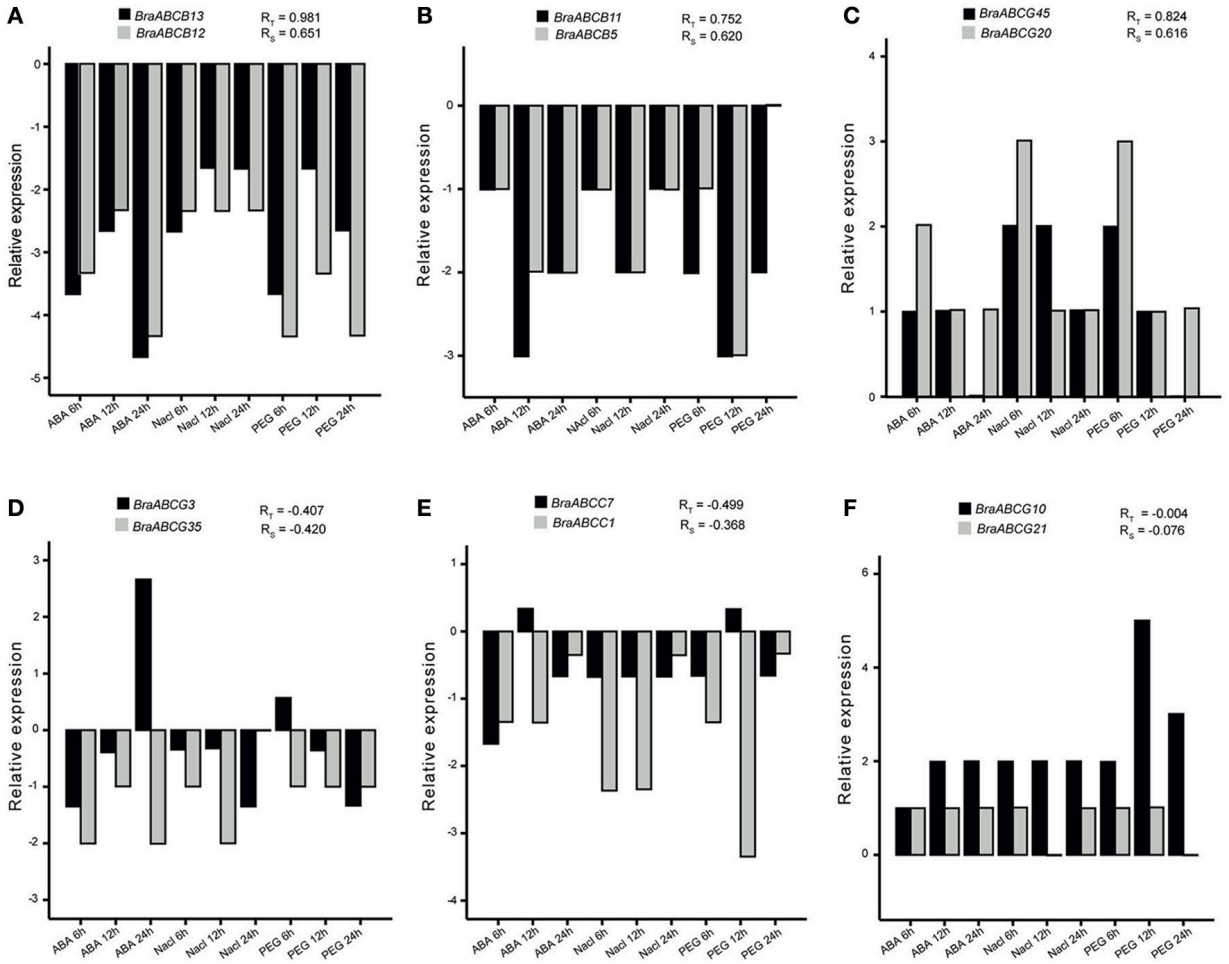
**Comparisons of the expression profiles of six pairs of syntenic BraABC paralogs across nine time points (in hours) after ABA, drought and salt stress treatments. (A–F)** Are the expression profiles of the selected six pairs of syntenic paralogs under different stress treatments at different time points, respectively. Each column represents the mean of three independent experiments, each with three replicates. The R_T_ and R_S_ are the Pearson correlation coefficients (PCCs) of paralog pairs measured by their expressions across different tissues and under different stress treatments, respectively.

We also investigated the expression patterns of syntenic BraABC paralog pairs under ABA, drought and salt stresses with qRT-PCR. Three pairs of conserved or sub-functional paralogs and three pairs of neo-functional paralogs were randomly selected to study their expression patterns. Primers for qRT-PCR are listed in Supplementary Table 4. As shown in Figure 8, a range of expression profiles of the selected BraABCs were observed after exposure to ABA, drought and salt stress. Some paralog pairs exhibited similar expression patterns, whereas others presented different expression profiles. The paralog pairs with positive expression correlations measured by PCCs across the five tissues also had positive correlations under the stress treatments (Figures 8A–C), which also indicated the functional conservation or sub-functionalization in these paralog pairs. Similarly, paralog pairs which exhibited negative correlations across the studied tissues also presented negative correlations under the stress treatments (Figures 8D–F), suggesting that these paralog pairs were neo-functional. On the whole, the expression correlations of the selected syntenic BraABC paralog pairs across the five tissues were consistent with those under the stress treatments.

### Interaction network analysis among BraABC proteins

**Figure 9:**
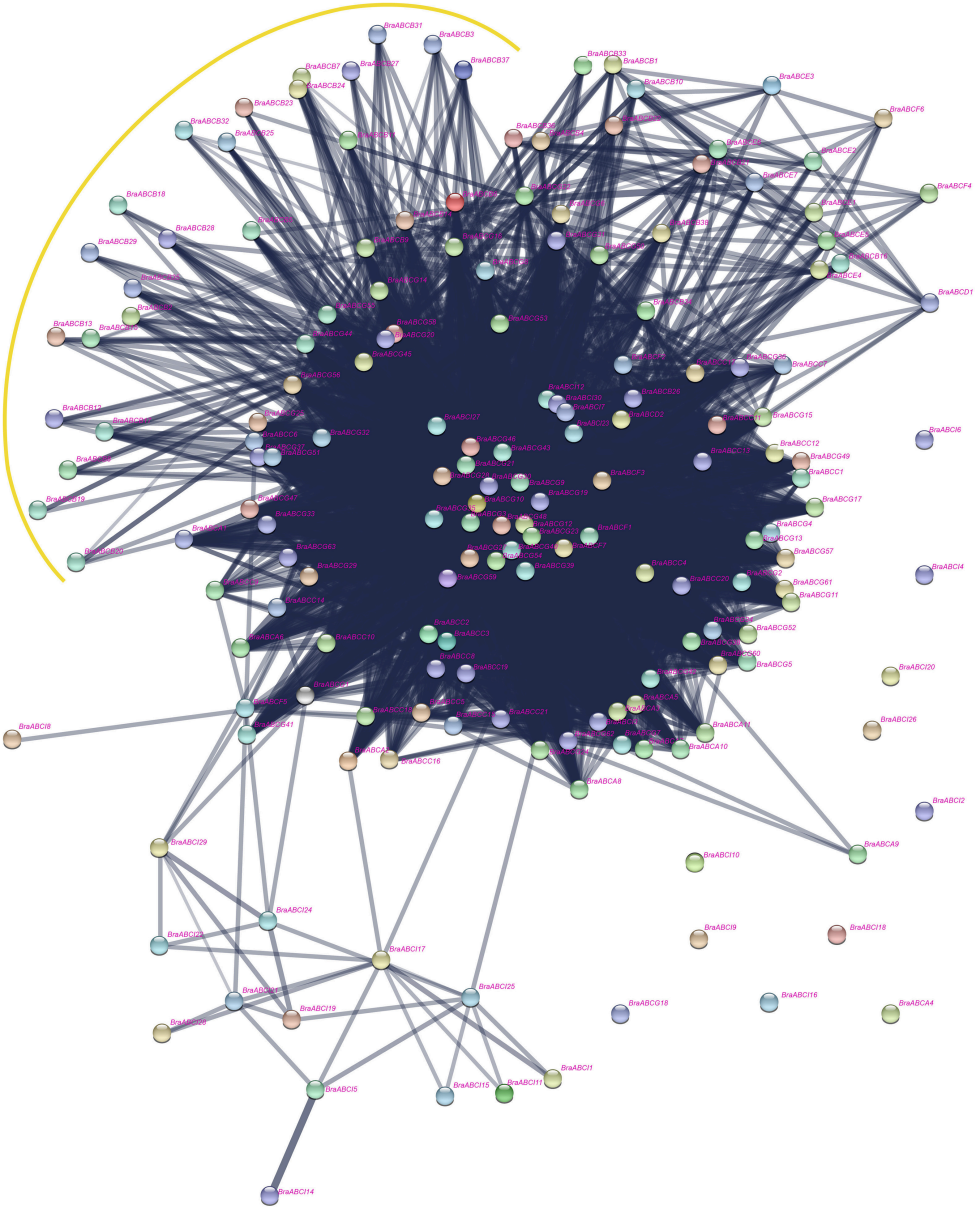
**Interaction network of BraABC proteins constructed using STRING software**.

The interaction network of BraABC proteins, including the functional and physical interactions, were examined using STRING software and the corresponding database to retrieve the protein interactions. As shown in Figure 9, a dense interaction network formed among the BraABC proteins. Most of the BraABC proteins were involved in interaction relations with other proteins, except for *BraABCA4, BraABCA9, BraABCG18*, and several ABCI proteins. Interaction relations not only existed among proteins within the same subfamily but also among proteins from different subfamilies. Though interacting frequently with proteins in other subfamilies, some ABCB proteins did not interact with proteins of ABCB, shown as being surrounded by the yellow curve in Figure 9. The majority of proteins located in the center of the network were ABCG proteins, indicating that these proteins had more complex interaction relations with other BraABC proteins. The gene dosage hypothesis predicts that genes will be preferentially retained if their products are dose sensitive, interacting either with other proteins or in networks (Thomas et al., 2006; Birchler and Veitia, 2007). Consequently, ABCG genes were probably more preferentially retained compared with genes of other subfamilies during evolution. Indeed, ABCG had the largest number of genes among all of the BraABC subfamilies.

## Discussion

Due to their sessile nature, plants have evolved complex movement systems to establish steep concentration gradients of solutes across cellular membranes to adapt to the variable environment. The particularly large complement of ABC proteins play a major role (George and Jones, 2012). ABC proteins are involved in various biological processes and are ubiquitous in all living organisms. To date, most research into the functions of ABCs has focused on *Arabidopsis* and rice, whereas studies are limited in terms of other non-model plants like *B. rapa* (Kang et al., 2011). In our research, we conducted comprehensive studies of ABCs in *B. rapa*, including whole genome-wide identification, evolutionary relationships, chromosomal locations, structural investigation, conservation after WGT and expression patterns in different tissues and under different stress treatments.

In this study, we systematically identified 179 BraABCs, representing 0.43% of the annotated protein coding genes in *B. rapa* (Wang et al., 2011). Gene duplication events are important to the rapid expansion and evolution of gene families. Furthermore, segmental duplication and tandem duplication are known to be major duplication modes for gene family expansion (Cannon et al., 2004). Previous studies revealed that *B. rapa* not only shared three paleo-polyploidy events with *Arabidopsis* but also underwent a further WGT event since its divergence from *Arabidopsis* 13 to 17 MYA (Town et al., 2006). In our study, the average duplication time of syntenic BraABC paralog pairs was 15.13 MYA, which was close to the recent WGT date of *B. rapa*. A total of 87 BraABCs were contained in the 76 pairs of BraABC syntenic paralogs among three subgenomes stemming from WGT. Simultaneously, 28 BraABCs were found to be located as 11 tandem arrays on chromosomes (Figure 3). Taken together, syntenic paralog analysis and chromosomal distribution revealed that the expansion of the ABC gene family in *B. rapa* could be attributed to the recent WGT and tandem duplication. Similarly, the expansion of other gene families in plants, such as the PG of *Populus*, the bZIP of apple and the ST of pear are also associated with WGD/segmental and tandem duplications (Yang et al., 2013; Li et al., 2015; Zhao et al., 2016).

The phylogenetic tree demonstrated that BraABCs can be divided into eight subfamilies (ABCA-ABCG and ABCI) (Supplementary Figure 1). All subfamilies, except ABCI, were clustered together in the phylogenetic tree constructed from ABCs of *B. rapa* and *Arabidopsis*, whereas the ABCI genes containing the NBD domain were scattered in the phylogenetic tree. This topology is similar to that of the phylogenetic tree constructed from the ABCs of maize (Pang et al., 2013). The dispersed distribution in the phylogenetic tree and non-uniform domain organization of ABCI genes indicated that the functions of ABCI are more divergent than other subfamilies. The subfamily classification was further supported by the results of domain organization, exon-intron structure and motif composition.

The evolution complexity of angiosperm genomes has been characterized by polyploidization through WGD followed by diploidization. Studies of *Arabidopsis* and its close relative *B. rapa* can provide fundamental insights into the evolutionary patterns of plant genomes. After triploidization, there is considerable gene loss (fractionation) in *B. rapa*, and is consistent with the widespread gene loss after WGD events in other eukaryotes (Sankoff et al., 2010). In theory, the number of ABCs in the newly formed hexaploid *B. rapa* genome would be three-fold as in *Arabidopsis*, whereas only 179 BraABCs were identified, indicating that some BraABCs were lost following WGT. The gene balance hypothesis predicts that genes whose products participate in macromolecular complexes, signaling networks or transcription, are more likely to be retained, avoiding network imbalances associated with the loss of one member (Thomas et al., 2006; Birchler and Veitia, 2007). In *B. rapa*, the ABCs were biased and preferentially retained. The preferential retentions of BraABCs were consistent with the gene balance hypothesis and demonstrate that BraABCs play key roles in the adaptation of *B. rapa*. In the interaction network of BraABC proteins (Figure 9), the ABCG proteins were highly connected with other BraABC proteins. Simultaneously, ABCG had the largest number of genes among all subfamilies, suggesting that ABCG genes were probably more preferentially retained during evolution. In turn, the preferential retention of ABCG genes supports the reliability of the gene balance hypothesis.

Evidence has demonstrated that ABC genes are involved in plant growth, development and stress tolerance. *AtABCB1*, a member of AtABC, can participate in auxin transport, and *AtABCB1* overexpressing plants develop longer hypocotyls (Sidler et al., 1998; Noh et al., 2001). *AtABCG25* is involved in abscisic acid transport and response (Kuromori et al., 2010). *OsABCC1*, a rice transporter, is involved in detoxification and reduce arsenic in rice grains (Song et al., 2014). Overexpression of *AtABCG36* improves drought and salt stress resistance in *Arabidopsis* (Kim et al., 2010). Gene expression patterns provide important clues for gene functions. Thus, we conducted expression analysis for all BraABCs in the roots, stems, leaves, flowers and siliques tissues using public RNA-seq data (Tong et al., 2013). Most of the BraABCs (92.2%) were expressed in at least one of the five tissues. Some BraABCs exhibited tissue-specific expression patterns (Figure 6B), indicating that they might play specific roles in the relevant tissues. *Cis*-elements in the promoters of genes can be bound by transcription factors and involved in gene expression regulation. To further understand the possible transcriptional regulation mechanisms of BraABCs, we scanned the common *cis*-regulatory elements, which were conserved in the promoter regions of all of the studied BraABCs. Among the identified 13 common *cis*-elements, WRKY71OS was responsive to ABA. However, in our qRT-PCR experiments, the expression profiles of some BraABCs and the presence of WRKY71OS in their promoters were not in good agreement under ABA treatment. Some BraABCs was down-regulated under ABA treatment or nearly unresponsive to ABA at some time points, such as *BrABCB13, BrABCB12, BrABCB11, BrABCB5*, and *BrABCG45* (Figure 8). This suggests that the induction of these BraABCs might result from the functions of a complex array of *cis*-elements, and some unidentified *cis*-elements might play a vital role in regulating the expression of these BraABCs under ABA treatment. Furthermore, this indicates that BraABCs have both shared, as well as distinct regulatory modules in response to ABA treatment. Moreover, of the identified common *cis*-elements, DOFCOREZM and GATABOX were related to carbon metabolism and molecular light switching, respectively, indicating that BraABCs probably participated in energy metabolism. Combining gene expression results with common *cis*-elements of BraABCs, we further confirmed the important roles of BraABCs during growth, development and stress response.

Altogether, 76 pairs of BraABC syntenic paralogs were identified among the three subgenomes. The average duplication time of the 76 pairs of paralogs was 15.13 MYA, which was consistent with the generated time of the three subgenomes (Wang et al., 2011). According to the ratio of non-synonymous to synonymous substitutions ω (= *Ka*/*Ks*), the selection type acting on the coding sequences can be measured. *Ka*/*Ks* ratios of 22 paralogs pairs were < 0.1, indicating that these paralog pairs were under strong purifying selection pressures. Furthermore, their functions were potentially more constrained with limited functional divergence occurring after duplication. It is worth noting that the ω ratios of 10 pairs of paralogs were > 1, representing positive selection and fast evolutionary rates in these BraABC paralogs at the protein level. This is different from other gene families in plants, such as PG of *Populus*, BURP of *Medicago* and ACD of tomato, which contain a few or even no paralog pairs undergoing positive selection (Yang et al., 2013; Li et al., 2016; Paul et al., 2016), and a relatively large percentage of BraABC paralogs pairs underwent positive selection in our study. We deduced genes of these paralog pairs might evolve some new functions to adjust to their living environment.

Four possible fates have been suggested for the evolution of gene duplication: functional conservation, sub-functionalization, neo-functionalization and pseudogenization (Lynch and Conery, 2000). Expression correlation analysis of syntenic BraABC paralog pairs across different tissues and under stress treatments could help reveal their functional roles in evolutionary fates. Our results indicate that all four fates turned up among the 76 pairs of paralogs. The high positive correlations of the 27 paralog pairs suggest the conservation of ancestral gene functions or sub-functionalization through the division of labor by retaining different parts of their ancestral functions. In contrast, 40 pairs of paralogs had small positive PCCs or negative PCCs, indicating that neo-functionalization and added functional diversity were likely to occur for the BraABC gene family. In particular, among the 9 pairs of paralogs undergoing pseudogenization, 8 pairs were composed of genes from two different subfamilies. Furthermore, ABCI genes were contained in each of these 8 pairs of paralogs. Interestingly, all of these ABCI genes had probably already become pseudogenes. Genes of ABCA-ABCG included multi-domains, whereas the ABCI genes generally contained only one domain, mainly NBD or TMD. We speculated that the ABCI genes in these eight paralog pairs were generated from the domain loss of genes in ABCA-ABCG. In short, the functions of genes in the BraABC gene family were enhanced and expanded through gene duplications. However, further functional analyses of BraABCs are still needed to determine which evolutionary fate the syntenic paralogs pairs undergo during the process of sequence and functional evolution.

In conclusion, we performed the first genome-wide analysis of the ABC gene family in *B. rapa*, and a total of 179 BraABCs were identified. Phylogenetic relationships, gene structures and *cis*-regulatory elements of BraABCs were studied in detail. Expression patterns of BraABCs were also characterized in different tissues and under different stresses. Furthermore, the conservation of BraABCs and the evolutionary fates of syntenic BraABC paralog pairs following WGT were studied. Our results could help to further investigate the biological functions of BraABCs in plant growth, development, and stress response.

## Author contributions

CY, WD, and XH conceived and designed the experiments. CY and SL performed the experiments. CY, WD, YL, and XH analyzed data. CY and XH wrote the manuscript. All authors read and approved the manuscript.

### Conflict of interest statement

The authors declare that the research was conducted in the absence of any commercial or financial relationships that could be construed as a potential conflict of interest.

## Abbreviations

ABC: ATP-binding cassette
ABCA: ABC subfamily A
ABCB: ABC subfamily B
ABCC: ABC subfamily C
ABCD: ABC subfamily D
ABCE: ABC subfamily E
ABCF: ABC subfamily F
ABCG: ABC subfamily G
ABCH: ABC subfamily H
ABCI: ABC subfamily I
ABCs: ABC genes
AtABC: Arabidopsis ABC
AtABCs: Arabidopsis ABC genes
BraABC: Brassica rapa ABC
BraABCs: Brassica rapa ABC genes
ML: maximum-likelihood
MYA: million years ago
NBD: nucleotide-binding domain
PCCs: Pearson correlation coefficients
PEG: polyethylene glycol
qRT-PCR: quantitative real-time PCR
TMD: transmembrane domains
WGD: whole genome duplication
WGT: whole genome triplication.
