# Supplementary material for: Genome-Wide Identification, Evolution, and Expression Analysis of the ATP-Binding Cassette Transporter Gene Family in Brassica rapa
Source: Front Plant Sci. 2017 Mar 17;8:349. doi: 10.3389/fpls.2017.00349 (PMC5355449; doi:10.3389/fpls.2017.00349)
Supplement: Supplementary file 1 [file Image1.PDF]

Supplementary Material

Genome-wide Identification, Evolution and Expression Analysis of the ATP-binding Cassette Transporter Gene Family in *Brassica rapa*

Chao Yan[1 †], Weike Duan[1, 2 †], Shanwu Lyu[1], Ying Li[1], Xilin Hou[1]*

[1]State Key Laboratory of Crop Genetics and Germplasm Enhancement, Key Laboratory of Biology and Germplasm Enhancement of Horticultural Crops in East China, Ministry of Agriculture, Nanjing Agricultural University, Nanjing 210095, China
[2]School of life science and food engineering, Huaiyin Institute of Technology, Huanan 223003, P.R. China

*** Correspondence:**
Prof. Xilin Hou
hxl@njau.edu.cn

Supplementary Figure 1-4

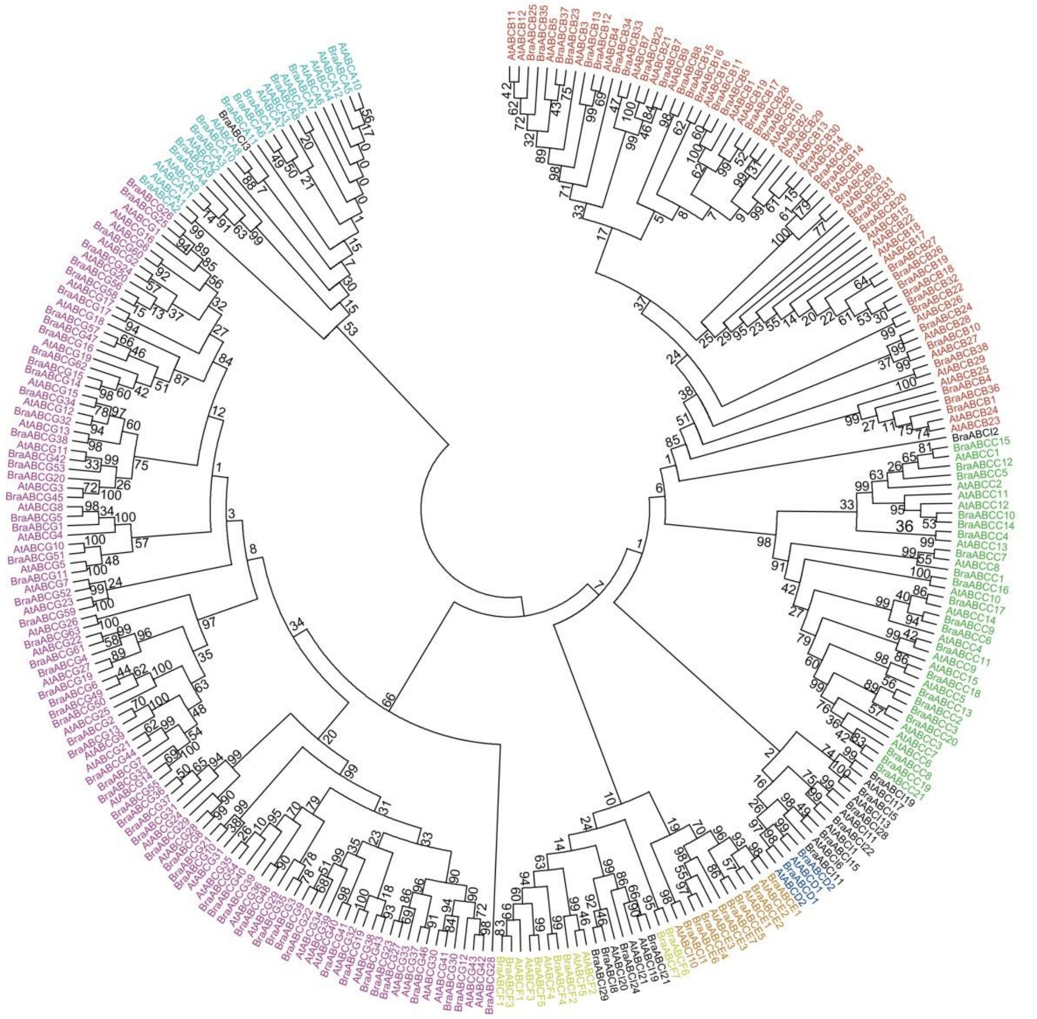

Supplementary Figure 1 Phylogenetic relationships of ABCs from *B. rapa* and *Arabidopsis*. The phylogenetic tree was constructed by MEGA6 using the maximum-likelihood (ML) procedure with 1000 bootstrap. Genes of different subfamilies are marked with different colors.

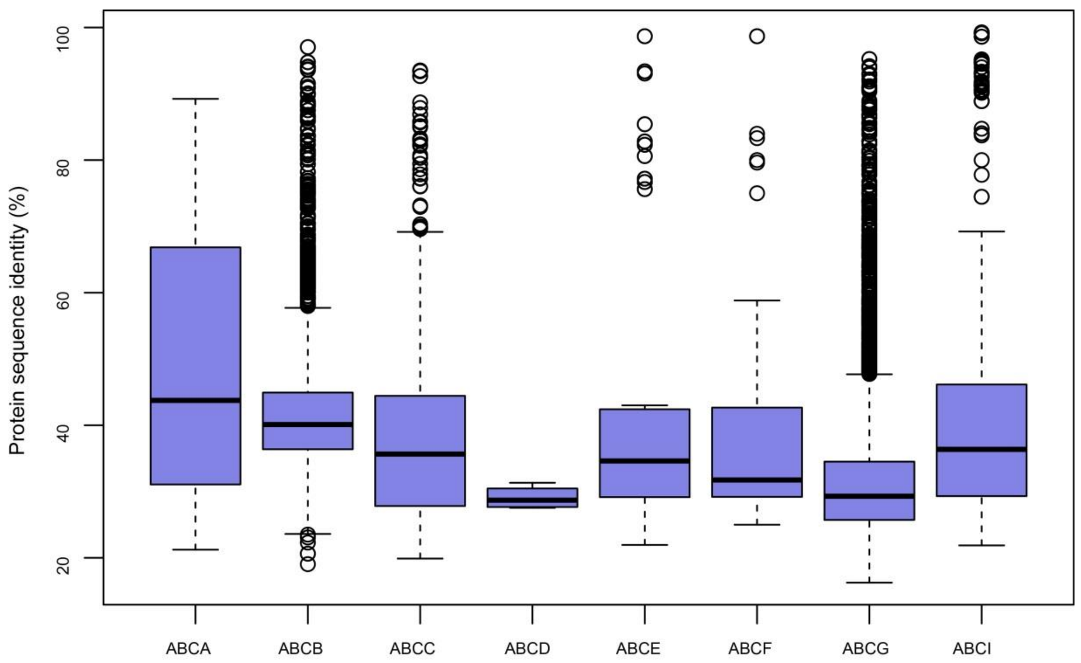

Supplementary Figure 2 Pairwise sequence identities of full-length BraABC proteins within each subfamily. The box plot shows the median (black line), interquartile range (box), and maximum and minimum scores (whiskers) of each data set. Outliers are shown as circles outside of the whiskers.

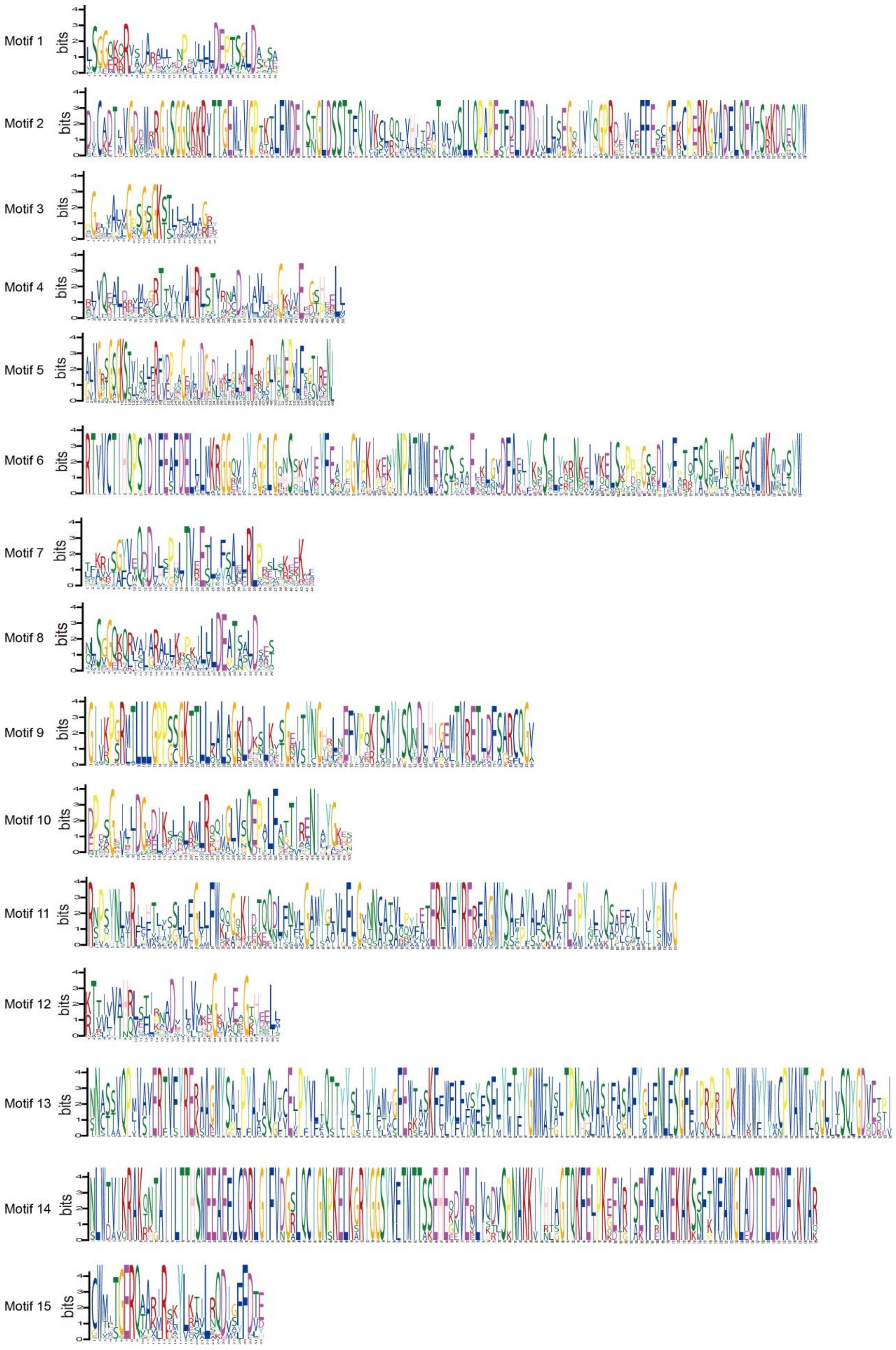

Supplementary Figure 3 Sequence logos of conserved motifs of BraABCs. The overall height of the stack indicates the degree of sequence conservation. The height of residues suggests the relative frequency of each residue at that position.

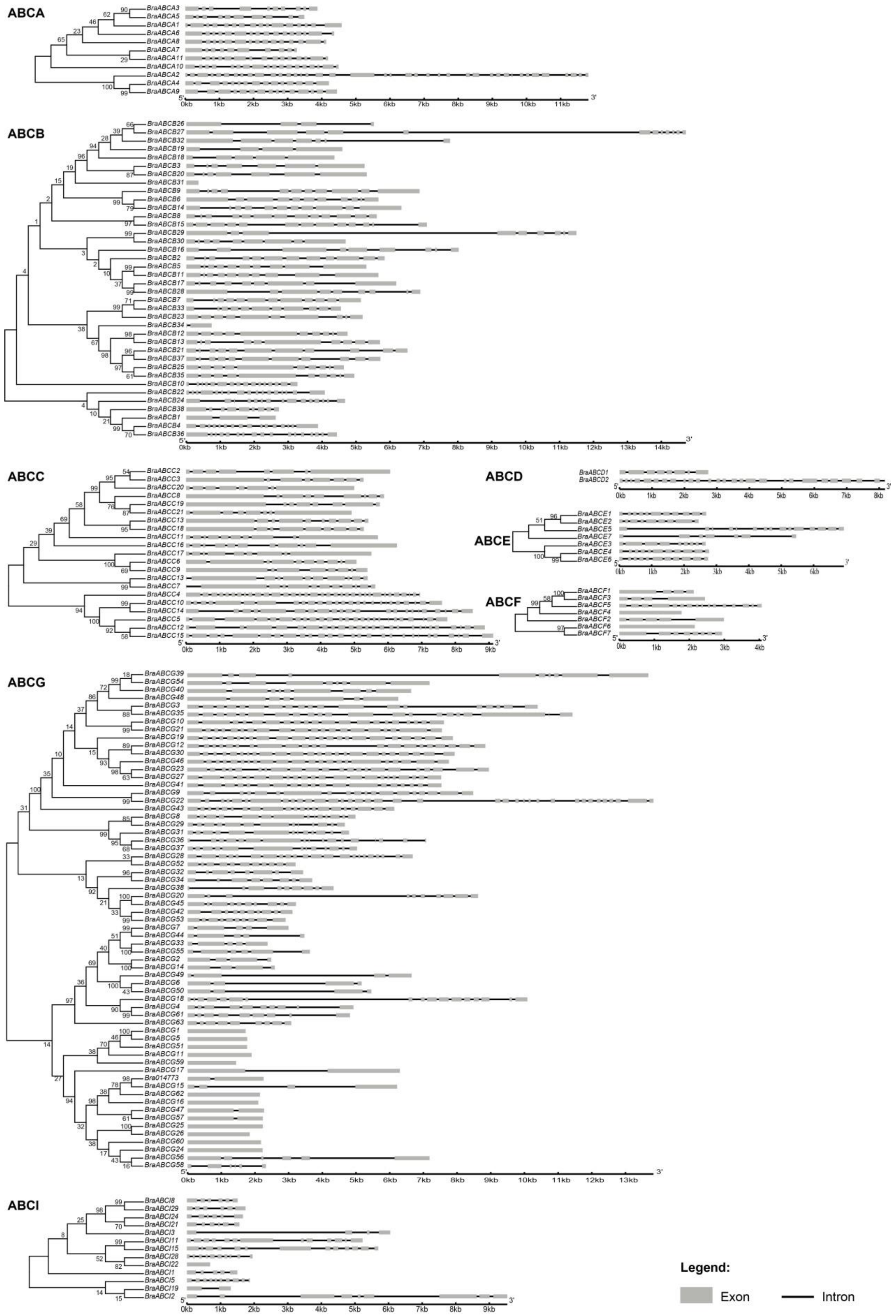

Supplementary Figure 4 The exon-intron organization of BraABCs in different subfamilies. Exons and introns are represented by gray boxes and black lines, respectively. The phylogenetic tree of each subfamily was constructed by MEGA6 using the maximum-likelihood (ML) procedure with 1000 bootstrap. For ABCI, its

phylogenetic tree was constructed only by ABCI genes containing NBD domain.
